# Tumor-derived exosomes induce N2 polarization of neutrophils to promote gastric cancer cell migration

**DOI:** 10.1186/s12943-018-0898-6

**Published:** 2018-10-06

**Authors:** Xu Zhang, Hui Shi, Xiao Yuan, Pengcheng Jiang, Hui Qian, Wenrong Xu

**Affiliations:** 1 Jiangsu Key Laboratory of Medical Science and Laboratory Medicine, School of Medicine, Jiangsu University, 301 Xuefu Road, Zhenjiang, 212013 Jiangsu China; 2 Institute of Digestive Diseases, The Affiliated People’s Hospital of Jiangsu University, 8 Dianli Road, Zhenjiang, 212002 Jiangsu China; 3 Zhenjiang Key Laboratory of Gastrointestinal Cancer, Jiangsu University, 301 Xuefu Road, Zhenjiang, 212013 Jiangsu China

**Keywords:** Exosome, Neutrophil, Gastric cancer, Pro-tumor, Activation, Autophagy

## Abstract

**Background:**

Exosomes are extracellular vesicles that mediate cellular communication in health and diseases. Neutrophils could be polarized to a pro-tumor phenotype by tumor. The function of tumor-derived exosomes in neutrophil regulation remains unclear.

**Methods:**

We investigated the effects of gastric cancer cell-derived exosomes (GC-Ex) on the pro-tumor activation of neutrophils and elucidated the underlying mechanisms.

**Results:**

GC-Ex prolonged neutrophil survival and induced expression of inflammatory factors in neutrophils. GC-Ex-activated neutrophils, in turn, promoted gastric cancer cell migration. GC-Ex transported high mobility group box-1 (HMGB1) that activated NF-κB pathway through interaction with TLR4, resulting in an increased autophagic response in neutrophils. Blocking HMGB1/TLR4 interaction, NF-κB pathway, and autophagy reversed GC-Ex-induced neutrophil activation. Silencing HMGB1 in gastric cancer cells confirmed HMGB1 as a key factor for GC-Ex-mediated neutrophil activation. Furthermore, HMGB1 expression was upregulated in gastric cancer tissues. Increased HMGB1 expression was associated with poor prognosis in patients with gastric cancer. Finally, gastric cancer tissue-derived exosomes acted similarly as exosomes derived from gastric cancer cell lines in neutrophil activation.

**Conclusion:**

We demonstrate that gastric cancer cell-derived exosomes induce autophagy and pro-tumor activation of neutrophils via HMGB1/TLR4/NF-κB signaling, which provides new insights into mechanisms for neutrophil regulation in cancer and sheds lights on the multifaceted role of exosomes in reshaping tumor microenvironment.

**Electronic supplementary material:**

The online version of this article (10.1186/s12943-018-0898-6) contains supplementary material, which is available to authorized users.

## Background

Neutrophils are important players in cancer development and progression [1–3]. In various cancers, neutrophils have been shown to promote carcinogenesis, growth and metastasis, angiogenesis, and immunosuppression. Neutrophils produce genotoxic substances such as reactive oxygen species (ROS) that can damage DNA in epithelial cells and initiate carcinogenesis. Neutrophils can also generate a wide spectrum of factors such as neutrophil elastase (NE) and prostaglandin E2 (PGE2) to promote tumor cell proliferation [4, 5]. In addition, neutrophils can promote tumor metastasis by enhancing tumor cell migration and invasion, degrading extracellular matrix, and promoting tumor cell colonization [6–8]. Meanwhile, neutrophils impair immunity to help tumor growth and metastasis [9–12]. Moreover, neutrophils produce a number of molecules such as matrix metalloproteinase-9 (MMP-9) and vascular endothelial growth factor (VEGF) to induce angiogenesis [13]. Increased neutrophil infiltration and elevated neutrophil/lymphocyte ratio (NLR) have been linked to disease progression and poor prognosis in cancer patients [14]. Targeting neutrophils to inhibit their pro-tumor function has shown therapeutic potential in mouse models [15]. Thus, better understanding of neutrophil regulation in cancer will provide new approaches for cancer diagnosis and therapy.

Accumulating studies suggest that tumor can induce a pro-tumor phenotype in neutrophils that, in turn, help tumor progression. The previous studies have shown that tumor cells produce oxysterol [16], C-X-C motif chemokine ligand 5 (CXCL5) [17], hyaluronan fragments (HA) [18], granulocyte-macrophage colony stimulating factor (GM-CSF) [19], and macrophage migration inhibitory factor (MIF) [20]. These factors induce pro-tumor activation of neutrophils, leading to increased tumor growth and metastasis. We have reported that IL-6 derived from tumor-resident mesenchymal stem cells induces neutrophil activation, resulting in enhanced angiogenesis and tumor metastasis [21]. Recently, Coffelt *et al.* demonstrated that IL-17 produced by tumor infiltrating ^γ^δ T cells could recruit, expand, and activate neutrophils to promote lung metastasis of breast cancer [22]. Nonetheless, mechanisms for the modulation of neutrophil phenotype and function in tumor milieu remain not fully characterized.

Exosomes are small lipid bilayer membrane vesicles of endocytic origin. Exosomes, as a novel mechanism of intercellular communication, can shuttle bioactive molecules from one cell to another, leading to the exchange of genetic information and reprogramming of recipient cells. Increasing evidence suggests that tumor cells release excessive amount of exosomes that promote tumor growth [23]. In addition, tumor-derived exosomes signal immune cells in tumor microenvironment, helping tumor cells escape immune surveillance and form pre-metastatic niche [24, 25]. We have recently shown that tumor cells interact with mesenchymal stem cells via exosomes to promote tumor growth, metastasis, and drug resistance [26–28]. However, the function of tumor-derived exosomes in neutrophil activation has not been well characterized.

In this study, we demonstrated that gastric cancer cells induced pro-tumor activation of neutrophils via exosomes. Gastric cancer cell-derived exosomes carried high mobility group box-1 (HMGB1) that interacted with toll-like receptor 4 (TLR4) to activate NF-κB and induce autophagy in neutrophils, which in turn promoted gastric cancer cell migration. Collectively, our findings indicate that exosomes represent a new regulator of neutrophil activation in gastric cancer.

## Results

### The conditioned medium from gastric cancer cells induces autophagy and pro-tumor activation of neutrophils

**Fig. 1:**
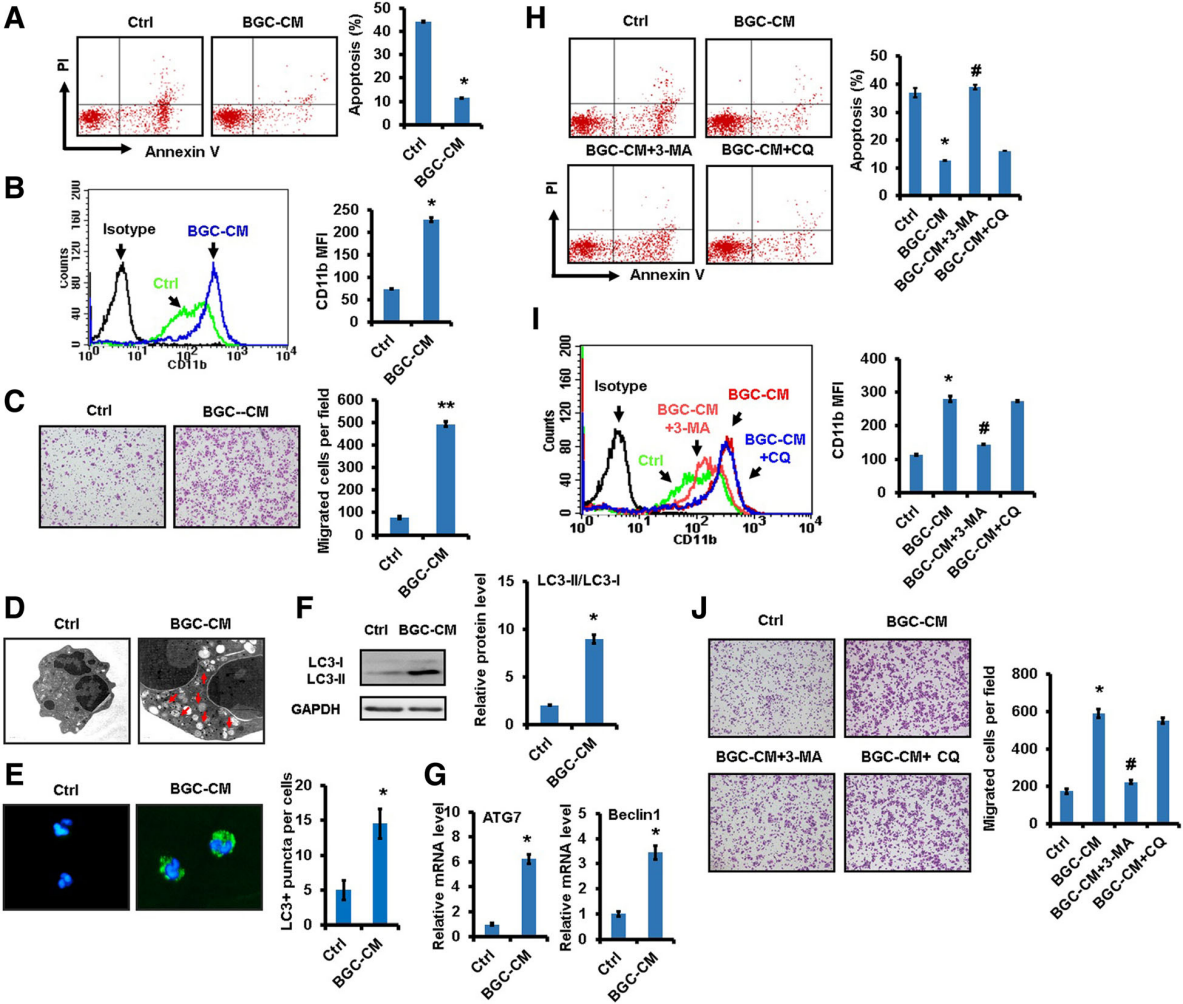
Gastric cancer cell-derived conditioned medium induced autophagy and pro-tumor activation of neutrophils. **a**. Flow cytometric analyses for apoptosis in neutrophils treated with or without conditioned medium from BGC-823 gastric cancer cells (BGC-CM). **b**. The expression of CD11b in BGC-CM-treated neutrophils was determined by flow cytometric analysis. **c**. Transwell migration assays for gastric cancer cells following treatment with supernatant from BGC-CM-treated neutrophils. **d**. Transmission electron microscopy analyses of autophagosomes (*arrows*) in neutrophils treated with BGC-CM. Scale bar=1 μm. **e**. Immunofluorescent staining of LC3-positive puncta in neutrophils treated with or without BGC-CM. Nuclei were counterstained by Hoechst 33342 (*blue*). **f**. Western blot assays for the expression of LC3-II in neutrophils incubated with conditioned medium from gastric cancer cells. **g**. The expression of *ATG7* and *BECN1* genes in neutrophils treated with conditioned medium from gastric cancer cells was determined by qRT-PCR. **h**. Neutrophils were pre-treated with autophagy inhibitors 3-MA or CQ followed by incubation with BGC-CM. The percentage of apoptotic neutrophils was determined by using flow cytometry. **i**. FACS analyses of CD11b expression in neutrophils treated with 3-MA and CQ prior to exposure to BGC-CM. **j**. Gastric cancer cells were incubated with the supernatants from BGC-CM-treated neutrophils that were pre-treated with or without autophagy inhibitors 3-MA or CQ and used for transwell migration assay. ^**^P<0.01 and ^*^P<0.05 compared to control (Ctrl); ^##^*P*<0.01 and ^#^*P*<0.05 compared to BGC-CM.

To investigate the role of gastric cancer cells in neutrophil phenotype and function, we treated neutrophils isolated from human peripheral blood with gastric cancer cell-derived conditioned medium (GC-CM) for 12 hours. Fluorescence-activated cell sorting (FACS) analyses showed that treatment with GC-CM inhibited the spontaneous apoptosis of neutrophils (Fig. 1a). In addition, GC-CM-treated neutrophils presented an increased expression of CD11b, an important molecule for neutrophil chemotaxis (Fig. 1b). Because tumors can modulate immune cells to acquire a pro-inflammatory phenotype, we determined the expression of inflammatory factors including IL-1β, IL-6, IL-8, oncostatin M (OSM), and TNFα in neutrophils. As shown in Additional file 1: Figure S1A, the expression of these inflammatory factors remarkably increased in GC-CM-treated neutrophils compared to controls. In addition, the expression of MMP9 and VEGF was also increased in GC-CM-treated neutrophils (Additional file 1: Figure S1B). GC-CM treatment inhibited ROS production while had minimal effect on the maturation state in neutrophils (Additional file 2: Figure S2A and B). We collected the supernatant from GC-CM-primed neutrophils and used it as chemoattracants for cell migration. The results of transwell migration assay showed that the supernatants from GC-CM-primed neutrophils promoted gastric cancer cell migration (Fig. 1c). Furthermore, GC-CM-primed neutrophils promoted gastric cancer cell proliferation and endothelial cell tube formation (Additional file 2: Figure S2C and D).

The promoting role of neutrophils in cancer progression has been linked to autophagic activation. Thus, we collected GC-CM-treated neutrophils and analyzed for autophagosomes. The results of transmission electron microscope (TEM) analyses showed that there were more autophagosomes in GC-CM-treated neutrophils than control cells (Fig. 1d). Immunofluorescent staining results confirmed the increase in the number of LC3-positive puncta in GC-CM-treated neutrophils compared to control cells (Fig. 1e). Moreover, western blot results showed an increase in LC3-II expression, an autophagosomal marker, in GC-CM-treated neutrophils (Fig. 1f). We then examined the expression of autophagy related 7 (*ATG7*) and Beclin 1 (*BECN1*) genes, important regulators of autophagosome formation, for cells treated with GC-CM. As expected, the increased expression of *ATG7* and *BECN1* was observed in GC-CM-treated neutrophils compared to controls (Fig. 1g). We next treated neutrophils with autophagy inhibitors and evaluated their phenotypic and functional changes in response to GC-CM treatment. As shown in Fig. 1h and i, the pre-treatment with autophagosome formation inhibitor 3-MA, but not autophagosome degradation inhibitor CQ, remarkably reversed the effects of GC-CM on neutrophil survival and CD11b expression. More importantly, the pre-treatment with 3-MA inhibited gastric cancer cell migration induced by GC-CM-primed neutrophils. This effect, however, was not seen for CQ (Fig. 1j). Taken together, these results demonstrate that gastric cancer cell-derived conditioned medium upregulates autophagy-related gene expression and induces autophagy in neutrophils, leading to pro-tumor activation of neutrophils.

### GC-CM activates NF-κB pathway in neutrophils

**Fig. 2:**
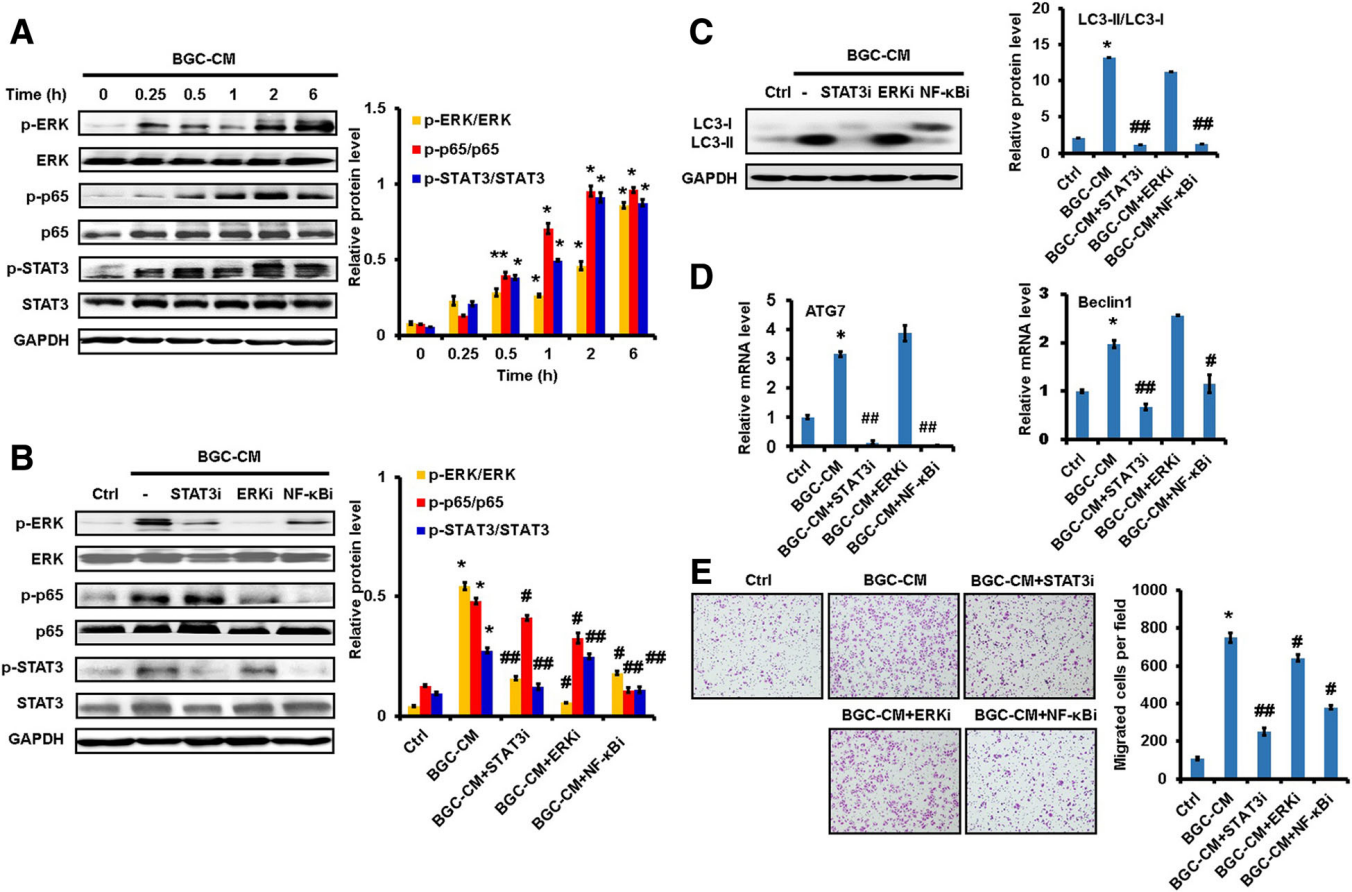
Gastric cancer cell-derived conditioned medium induced pro-tumor activation of neutrophils through NF-κB pathway. **a**. Western blot assays for the expression of p-p65, p-STAT3, and p-ERK in neutrophils treated with GC-CM for different times. **b-e**. Neutrophils were pre-treated with inhibitors for NF-κB, STAT3, and ERK pathways followed by incubation with BGC-CM. The expression of phosphorylated NF-κB, STAT3, and ERK in neutrophils was determined by western blot (**b**). Western blot assays for LC3-II expression in neutrophils (**c**). QR-PCR analyses of *ATG7* and *BECN1* expression in neutrophils (**d**). Gastric cancer cells were incubated with the supernatants from neutrophils and used for transwell migration assay (**e**). ^**^P<0.01 and ^*^P<0.05 compared to control; ^##^*P*<0.01 and ^#^*P*<0.05 compared to BGC-CM

To clarify mechanisms for the pro-tumor activation of neutrophils, we treated neutrophils with GC-CM and determined the responses of various pathways. GC-CM treatment increased the expression of phosphorylated p65 (p-p65), p-STAT3, and p-ERK in neutrophils in a time-dependent manner (Fig. 2a). We also found that the expression of p-p38 and p-Akt was modestly increased in GC-CM-treated neutrophils (Additional file 2: Figure S2E). Notably, NF-κB inhibitor reversed GC-CM-induced activation of STAT3 and ERK pathways (Fig. 2b). In consistent with these observations, NF-κB inhibitor completely blocked GC-CM-induced expression of inflammatory factors while only partial inhibition was noted for STAT3 and ERK inhibitors (Additional file 3: Figure S3), suggesting a dominant role of NF-κB pathway. In particular, the pre-treatment of neutrophils with NF-κB inhibitor reversed GC-CM-induced expression of LC3-II, supporting that NF-κB pathway is critically involved in GC-CM-induced autophagy in neutrophils (Fig. 2c). The increased expression of *ATG7* and *BECN1* in GC-CM-treated neutrophils was also blocked by NF-κB inhibitor (Fig. 2d). Finally, GC-CM-primed neutrophil-induced gastric cancer cell migration was blocked by NF-κB inhibitor (Fig. 2e). These results suggest that gastric cancer cell-derived conditioned medium induces a pro-tumor phenotype in neutrophils through activating NF-κB pathway.

### Gastric cancer cell-derived exosomes induce autophagy and pro-tumor activation of neutrophils

**Fig. 3:**
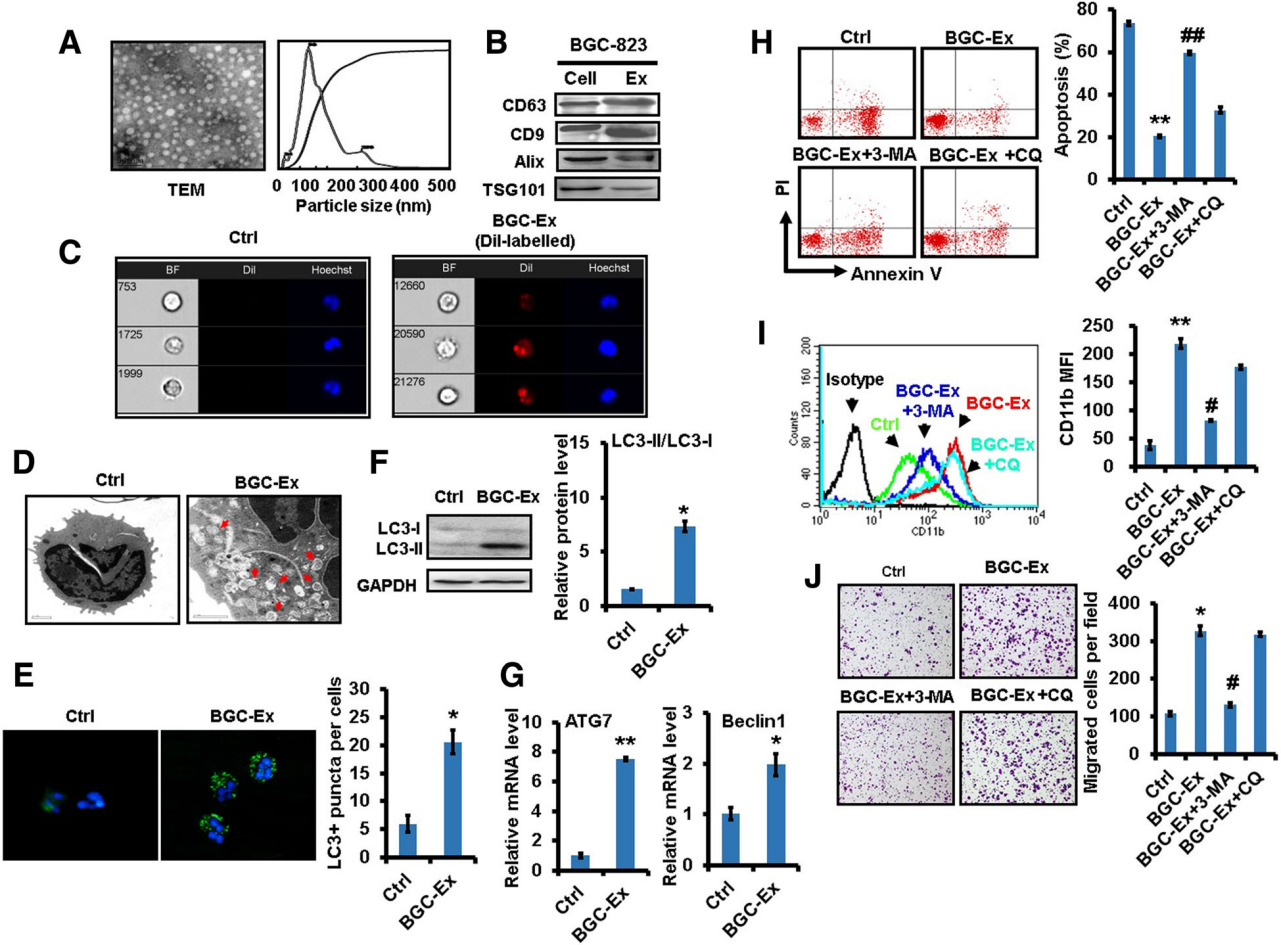
Gastric cancer cell-derived exosomes induced autophagy to promote pro-tumor activation of neutrophils. **a**. The morphology (*left*) and size (*right*) of gastric cancer cell-derived exosomes were determined by TEM and NTA, respectively. **b**. The expression of exosomal markers CD9 and CD63 was determined by western blot. **c**. The internalization of CM-Dil-labelled exosomes (*red*) by neutrophils was determined by Imaging flow cytometry. Nuclei were counterstained with Hoechst 33342 (*blue*). BF, bright field. **d**. The presence of autophagosomes (*arrows*) in neutrophils treated with gastric cancer cell-derived exosomes (BGC-Ex) was determined by TEM. Scale bar=1 μm. **e**. Immunofluorescent staining of LC3-positive puncta (*green*) in BGC-Ex-treated neutrophils. **f**. The expression of LC3-II in BGC-Ex-treated neutrophils was confirmed by western blot. **g**. QRT-PCR analyses of *ATG7* and *BECN1* expression in BGC-Ex-treated neutrophils. **h-j**. Neutrophils were pre-treated with autophagy inhibitors 3-MA and CQ followed by treatment with BGC-Ex. FACS analyses of spontaneous apoptosis in neutrophil were shown (**h**). CD11b expression in neutrophils was determined by FACS analyses (**i**). Gastric cancer cells were incubated with the supernatants from neutrophils and used for transwell migration assay (**j**). ^**^P<0.01 and ^*^P<0.05 compared to control (Ctrl); ^##^*P*<0.01 and ^#^*P*<0.05 compared to BGC-Ex

Gastric cancer cells can release exosomes to activate mesenchymal stem cells and macrophages [27, 29]. We next determined whether or not tumor-derived exosomes are involved in the pro-tumor activation of neutrophils. We found that the gastric cancer cell-derived exosomes (GC-Ex) displayed sphere-like morphology with a diameter ~100 nm and expressed the exosomal markers CD9 and CD63 (Fig. 3a and b). To determine the uptake of GC-Ex by neutrophils, GC-Ex was labeled with membrane-bound fluorescent dye CM-Dil and added to neutrophil cultures. Imaging flow cytometry results showed that GC-Ex was efficiently internalized by neutrophils (Fig. 3c). We next investigated the ability of GC-Ex to induce autophagy in neutrophils. The results of TEM and immunofluorescent staining showed that the number of autophagosomes was increased in GC-Ex-treated neutrophils compared to control cells (Fig. 3d and e). GC-Ex treatment also increased LC3-II expression as well as *ATG7* and *BECN1* expression in neutrophils (Fig. 3f and g). Furthermore, GC-Ex protected neutrophils against spontaneous apoptosis and induced CD11b expression in neutrophils. To demonstrate which step in the process of autophagy GC-Ex may act on, we pre-treated neutrophils with autophagosome formation inhibitor 3-MA or autophagosome degradation inhibitor CQ followed by incubation with GC-Ex. The effects of GC-Ex on neutrophil apoptosis and CD11b expression were blocked by 3-MA. In contrast, no effect was noted for CQ (Fig. 3h and i). Finally, the supernatant from GC-Ex-treated neutrophils promoted gastric cancer cell migration. This increase was blocked by 3-MA but not CQ (Fig. 3j), suggesting that GC-Ex trigger autophagosome formation in neutrophils. Furthermore, GC-Ex treatment inhibited ROS production while had minimal effect on the maturation state in neutrophils (Additional file 4: Figure S4A and B). GC-Ex-primed neutrophils promoted gastric cancer cell proliferation and endothelial cell tube formation (Additional file 4: Figure S4C and D). The expression of MMP9 and VEGF was upregulated in GC-Ex-treated neutrophils (Additional file 4: Figure S4E). These findings suggest that neutrophils can uptake GC-Ex to induce autophagy and pro-tumor activation.

### GC-Ex induces neutrophil activation through NF-κB pathway

**Fig. 4:**
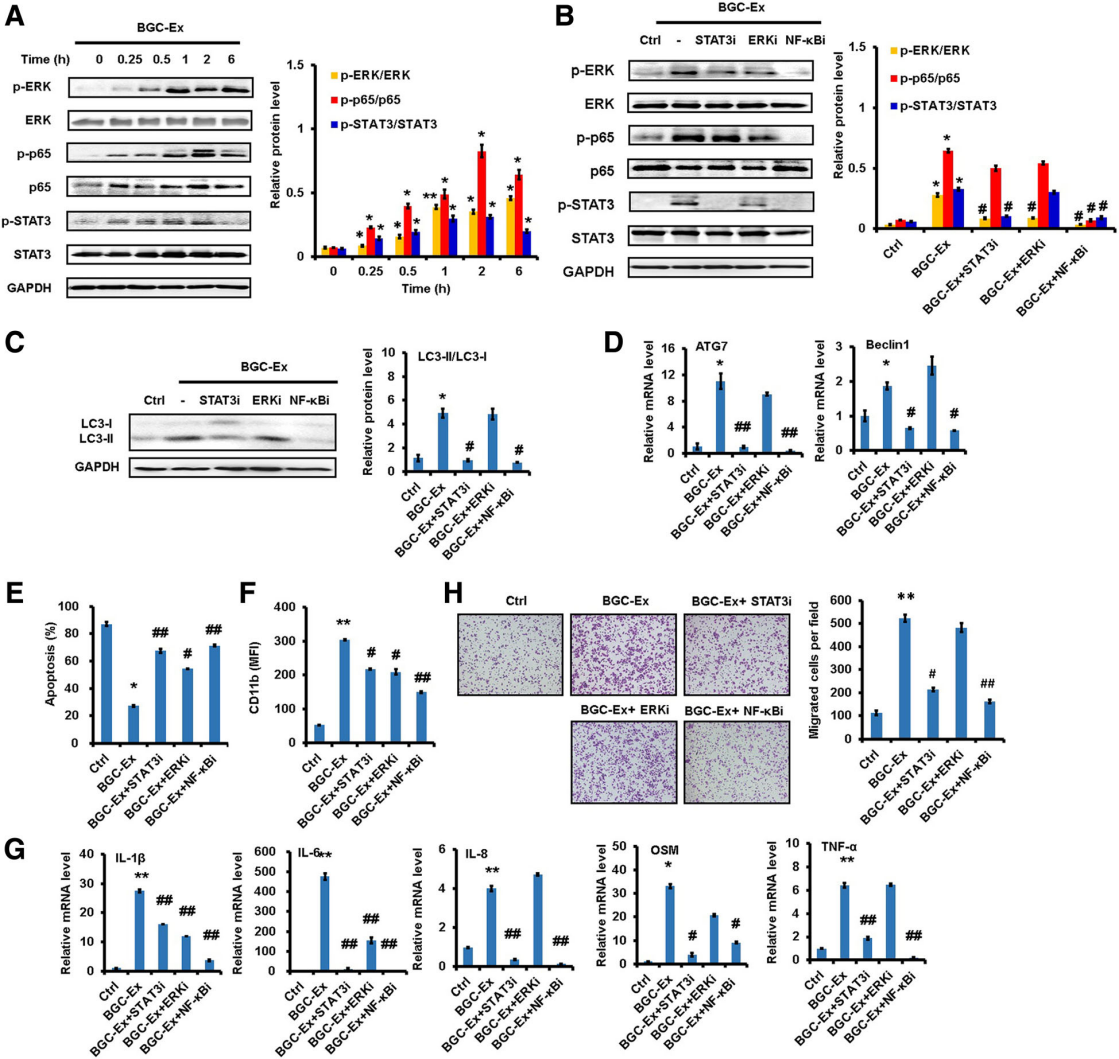
Gastric cancer cell-derived exosomes induced pro-tumor activation of neutrophils through NF-κB pathway. **a**. Western blot assays for the expression of p-p65, p-STAT3, and p-ERK in neutrophils treated with GC-Ex for different times. **b-h**. Neutrophils were pre-treated with inhibitors for NF-κB, STAT3, and ERK pathways followed by incubation with BGC-Ex. The expression of phosphorylated NF-κB, STAT3, and ERK in neutrophils was determined by western blot (**b**). Western blot assays for the expression of LC3-II in neutrophils (**c**). The expression of *ATG7* and *BECN1* genes in neutrophils was determined by qRT-PCR (**d**). Flow cytometric analyses for apoptosis in neutrophils (**e**). The expression of CD11b in neutrophils (**f**). QRT-PCR analyses of pro-inflammatory factor gene expression (IL-1β, IL-6, IL-8, OSM, and TNFα) in neutrophils (**g**).Transwell migration assays for gastric cancer cells following treatment with supernatant from neutrophils (**h**). ^**^P<0.01 and ^*^P<0.05 compared to control (Ctrl); ^##^*P*<0.01 and ^#^*P*<0.05 compared to BGC-Ex

Neutrophils were exposed to GC-Ex and the activation state of various pathways were analyzed. The results of western blot showed that the expression of p-p65, p-STAT3, and p-ERK was increased in GC-Ex-treated neutrophils compared to untreated controls (Fig. 4a). The expression of phosphorylated p-p38 and p-Akt was also modestly increased in neutrophils after GC-Ex treatment (Additional file 4: Figure S4E). The pre-treatment with NF-κB inhibitor blocked GC-Ex-induced activation of STAT3 and ERK in neutrophils (Fig. 4b). In addition, NF-κB inhibitor blocked GC-Ex-induced LC3-II production as well as *ATG7* and *BECN1* expression in neutrophils (Fig. 4c and d). Similarly, GC-Ex-induced decrease of spontaneous apoptosis and increase of CD11b in neutrophils were reversed by NF-κB inhibitor (Fig. 4e and f). NF-κB inhibitor also blocked GC-Ex-induced the expression of inflammatory factors in neutrophils (Fig. 4g). Moreover, NF-κB inhibitor impaired the migration-promoting effect caused by exposure of gastric cancer cells to the supernatant from GC-Ex-treated neutrophils (Fig. 4h). In summary, these results suggest that GC-Ex induces autophagy and pro-tumor activation of neutrophils through NF-κB pathway.

### GC-Ex induces neutrophil activation through the interaction between high mobility group box-1 and Toll-like receptor 4

Exosomes can induce signaling pathway activation in recipient cells by delivering bioactive molecules. To investigate the molecules in GC-Ex that induce neutrophil activation, we treated GC-Ex with proteinase and evaluated the effects of digested exosomes on neutrophil activation. Compared to undigested GC-Ex, the treatment of proteinase-digested GC-Ex decreased LC3-II expression as well as *ATG7* and *BECN1* expression in neutrophils (Additional file 5: Figure S5A and B). In similar, GC-Ex-induced activation of NF-κB, STAT3, and ERK pathways in neutrophils was also impaired by proteinase treatment (Additional file 5: Figure S5C). Moreover, the treatment with proteinase decreased GC-Ex-induced expression of inflammatory factors in neutrophils compared to undigested GC-Ex (Additional file 5: Figure S5D). Thus, we focused on exosomal proteins in the subsequent studies.

**Fig. 5:**
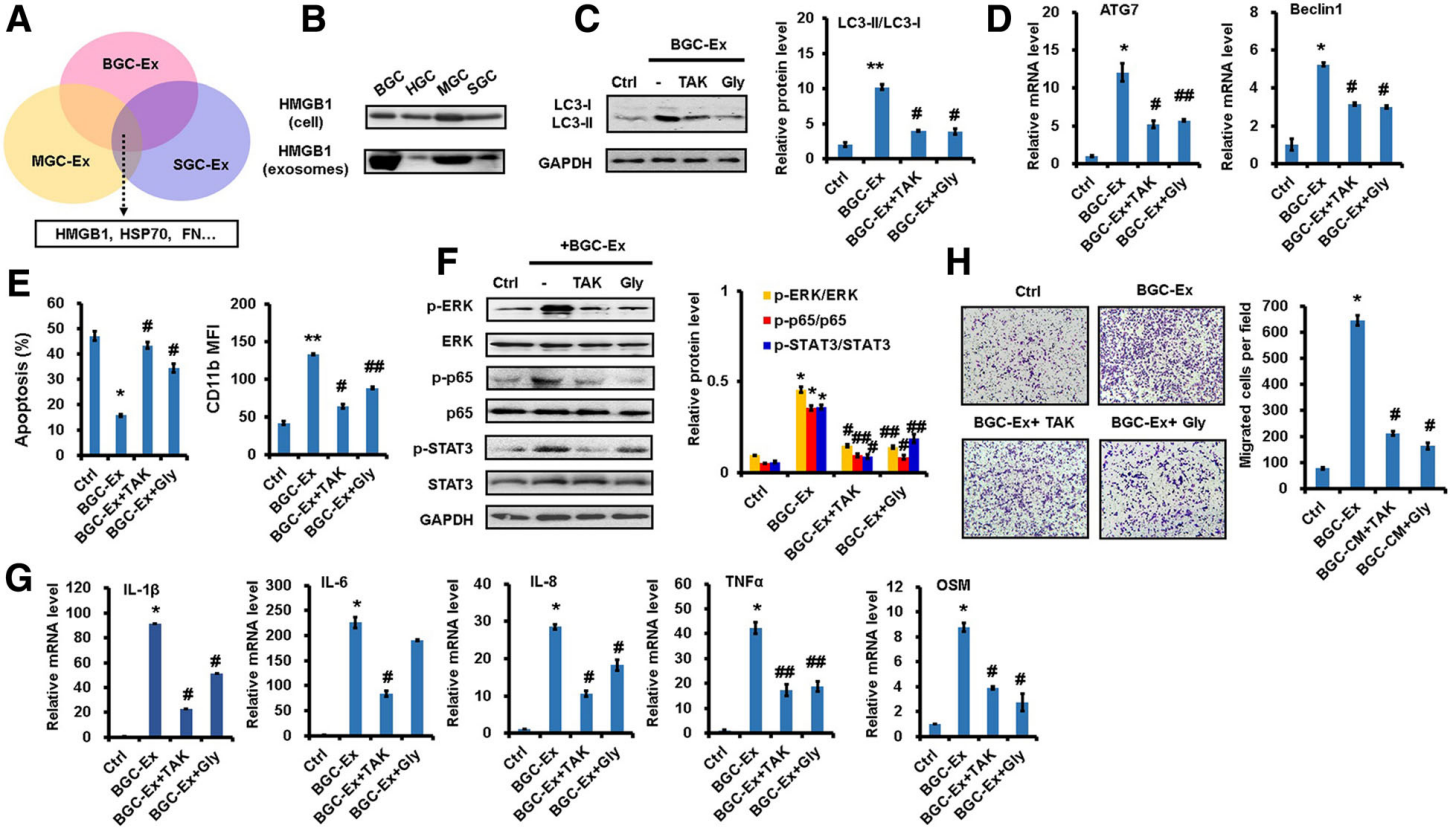
Gastric cancer cell-derived exosomes induced neutrophil activation through interaction of HMGB1 and TLR4. **a**. LC-MS/MS proteomic analyses for exosomes from gastric cancer cell lines (BGC-823, MGC80-3, and SGC-7901). **b**. The expression of HMGB1 in gastric cancer cell-derived exosomes was verified by western blot. **c-h**. Neutrophils were pre-treated with HMGB1 antagonist (Gly) or TLR4 inhibitor (TAK) followed by treatment with BGC-Ex. Western blot assays for the expression of LC3-II in neutrophils (**c**). The expression of *ATG7* and *BECN1* genes in neutrophils was determined by qRT-PCR (**d**). Flow cytometric analyses for apoptosis and CD11b expression in neutrophils (**e**). The expression of phosphorylated NF-κB, STAT3, and ERK in neutrophils was determined by western blot (**f**). QRT-PCR analyses of pro-inflammatory factor gene expression (IL-1β, IL-6, IL-8, OSM, and TNFα) in neutrophils (**g**).Transwell migration assays for gastric cancer cells following treatment with supernatant from neutrophils (**h**). ^**^P<0.01 and ^*^P<0.05 compared to control (Ctrl); ^##^*P*<0.01 and ^#^*P*<0.05 compared to BGC-Ex

We then tested the roles of exosomes from three other gastric cancer cell lines. Consistent with that observed in exosomes from BGC-823 gastric cancer cells, the exosomes from HGC-27, MGC-803, and SGC-7901 cells also induced autophagy and pro-tumor activation in neutrophils (Additional file 6: Figure S6). To identify protein(s) that mediate the roles of exosomes in neutrophil activation, we performed a proteomic analysis for exosomes from three gastric cancer cell lines (Additional file 7: Table S3). Several proteins of interest were identified such as high mobility group Box-1 (HMGB1), heat-shock protein 70 (HSP70), fibronectin (FN) (Fig. 5a). The results of western blot confirmed HMGB1 expression in GC-Ex from all of the tested gastric cancer cell lines (Fig. 5b). HMGB1 have previously shown to play important roles in inflammation and cancer. Thus, we chose HMGB1 as the target for further study.

We next investigated the role of exosomal HMGB1 in neutrophil activation. Neutrophils were treated with HMGB1 antagonist and then exposed to GC-Ex. HMGB1 antagonist reversed GC-Ex-induced increases in LC3-II expression and *ATG7* and *BECN1* expression in neutrophils compared to GC-Ex alone (Fig. 5c and d). In addition, GC-Ex-induced apoptotic inhibition as well as increases of CD11b expression in neutrophils were inhibited by HMGB1 antagonist (Fig. 5e). Moreover, HMGB1 antagonist inhibited GC-Ex-induced activation of NF-κB pathway and expression of inflammatory factors in neutrophils (Fig. 5f and g). Finally, the promoting effect of supernatant from GC-Ex-treated neutrophils on gastric cancer cell migration was impaired by HMGB1 antagonist (Fig. 5h).

HMGB1 activates NF-κB pathway by interacting with toll-like receptor (TLR) and receptor for advanced glycation end products (RAGE). We thus examined the effects of TLR2, TLR4, and RAGE inhibitors on the observed roles of GC-Ex in neutrophils. As expected, TLR4 inhibitor blocked GC-Ex-induced autophagy and NF-κB pathway activation in neutrophils (Fig. 5c, d and f). TLR4 inhibitor decreased the expression of inflammatory factors in GC-Ex-treated neutrophils and impaired its gastric cancer cell migration-promoting effect (Fig. 5g and h; Additional file 5: Figure S5E). However, the inhibitors for TLR2 and RAGE showed minimal effects (data not shown). Taken together, these results suggest that GC-Ex delivers HMGB1 to neutrophils and induces pro-tumor activation through TLR4/NF-κB signaling.

### Gastric cancer tissue-derived exosomes induce autophagy and pro-tumor activation of neutrophils

**Fig. 6:**
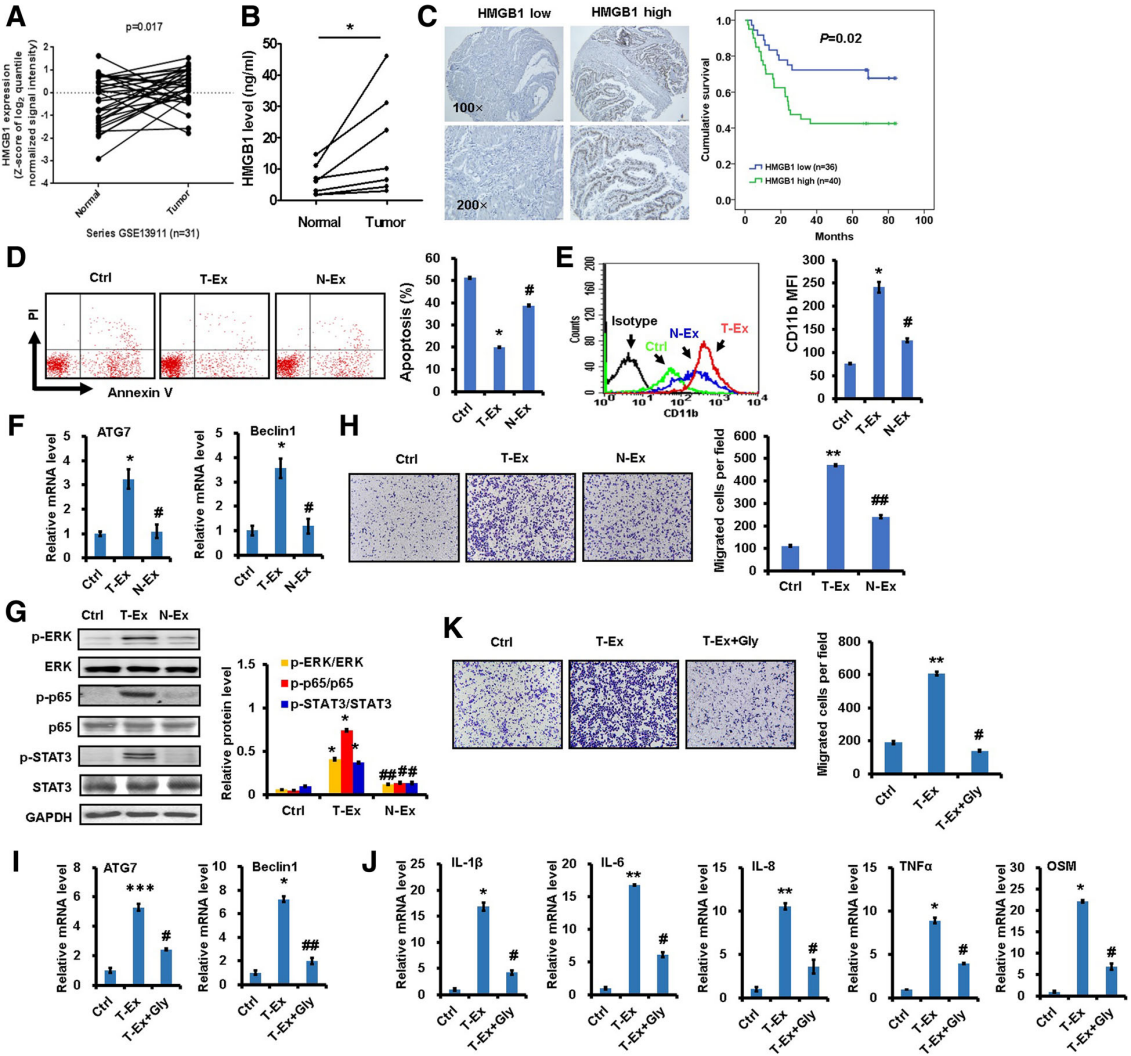
Gastric cancer tissue-derived exosomes induced pro-tumor activation of neutrophils. **a**. *In silicon* analyses of HMGB1 expression in paired gastric cancer and non-cancerous tissues from GEO dataset (GSE13911). **b**. The expression of HMGB1 in gastric cancer tissues and paired non-cancerous tissues was measured by ELISA (n=9). **c**. Tissue microarray analyses of HMGB1 expression in paired gastric cancer tissues and non-cancerous tissues (n=76). Survival time of gastric cancer patients was analyzed by Kaplan-Meier analysis. **d-e**. The percentage of apoptotic cells (**d**) and CD11b expression (**e**) in neutrophils treated with gastric cancer tissue-derived exosomes (T-Ex) and non-cancerous tissue-derived exosomes (N-Ex) were determined by flow cytometry. **f**. QRT-PCR analyses of *ATG7* and *BECN1* expression in neutrophils treated with T-Ex and N-Ex. **g**. Western blot assays for the activation of NF-κB, STAT3, and ERK pathways in neutrophils treated with T-Ex and N-Ex. **h**. Gastric cancer cells were incubated the supernatants from T-Ex- and N-Ex-treated neutrophils and used for transwell migration assay. **i-k**. Neutrophils were treated with T-Ex in the presence or absence of HMGB1 antagonist (Gly). The expression of *ATG7* and *BECN1* (**i**) and inflammatory factors (**j**) is measured by qRT-PCR. **k**. Transwell migration assays for gastric cancer cells following treatment with supernatant from neutrophils. ^*^P<0.05 compared to control (Ctrl); ^##^*P*<0.01 and ^#^*P*<0.05 compared to T- Ex

*In silico* gene expression analysis (Dataset GSE13911 retrieved from Gene Expression Omnibus) showed remarkably increased *HMGB1* expression in gastric cancer tissues compared to the paired non-cancerous tissues (Fig. 6a). Therefore, we measured HMGB1 in paired gastric cancer tissues and non-cancerous tissues by ELISA. The expression of HMGB1 was increased in gastric cancer tissues compared to non-cancerous tissues (Fig. 6b). To determine whether increased HMGB1 expression is associated with clinical outcomes, HMGB1 was analyzed in the cancer and non-cancerous tissues from gastric cancer patients by using tissue microarray. We found that a higher level of HMGB1 in gastric cancer patients predicted poorer prognosis (Fig. 6c). In addition, we found that HMGB1 expression levels were higher in exosomes from the culture supernatants of gastric cancer tissues than non-cancerous tissues and in exosomes from the serum samples of gastric cancer patients than healthy controls (Additional file 8: Figure S7).

To confirm our findings from gastric cancer cell lines, we tested neutrophil activation by using exosomes from matched gastric cancer and non-cancerous tissues. Similar to GC-Ex, exosomes from gastric cancer tissues prevented the apoptosis of neutrophils and induced CD11b expression in neutrophils (Fig. 6d and e). Gastric cancer tissue-derived exosomes also induced higher *ATG7* and *BECN1* expression (Fig. 6f) and NF-κB activation (Fig. 6g) in neutrophils than non-cancerous tissue-derived exosomes. Neutrophils treated with exosomes from gastric cancer tissues promoted gastric cancer cell migration more efficiently (Fig. 6h). In addition, HMGB1 antagonist reversed gastric cancer tissue-derived exosomes-induced increases of *ATG7* and *BECN1* expression in neutrophils (Fig. 6i). Moreover, gastric cancer tissue-derived exosomes-induced expression of inflammatory factors in neutrophils was inhibited by HMGB1 antagonist (Fig. 6j). Finally, the promoting effect of supernatant from gastric cancer tissue-derived exosomes-treated neutrophils on gastric cancer cell migration was impaired by HMGB1 antagonist (Fig. 6k). Taken together, these findings indicate that gastric cancer tissue-derived exosomes could induce autophagy and pro-tumor activation of neutrophils.

### *HMGB1* knockdown impairs GC-Ex-induced neutrophil activation

**Fig. 7:**
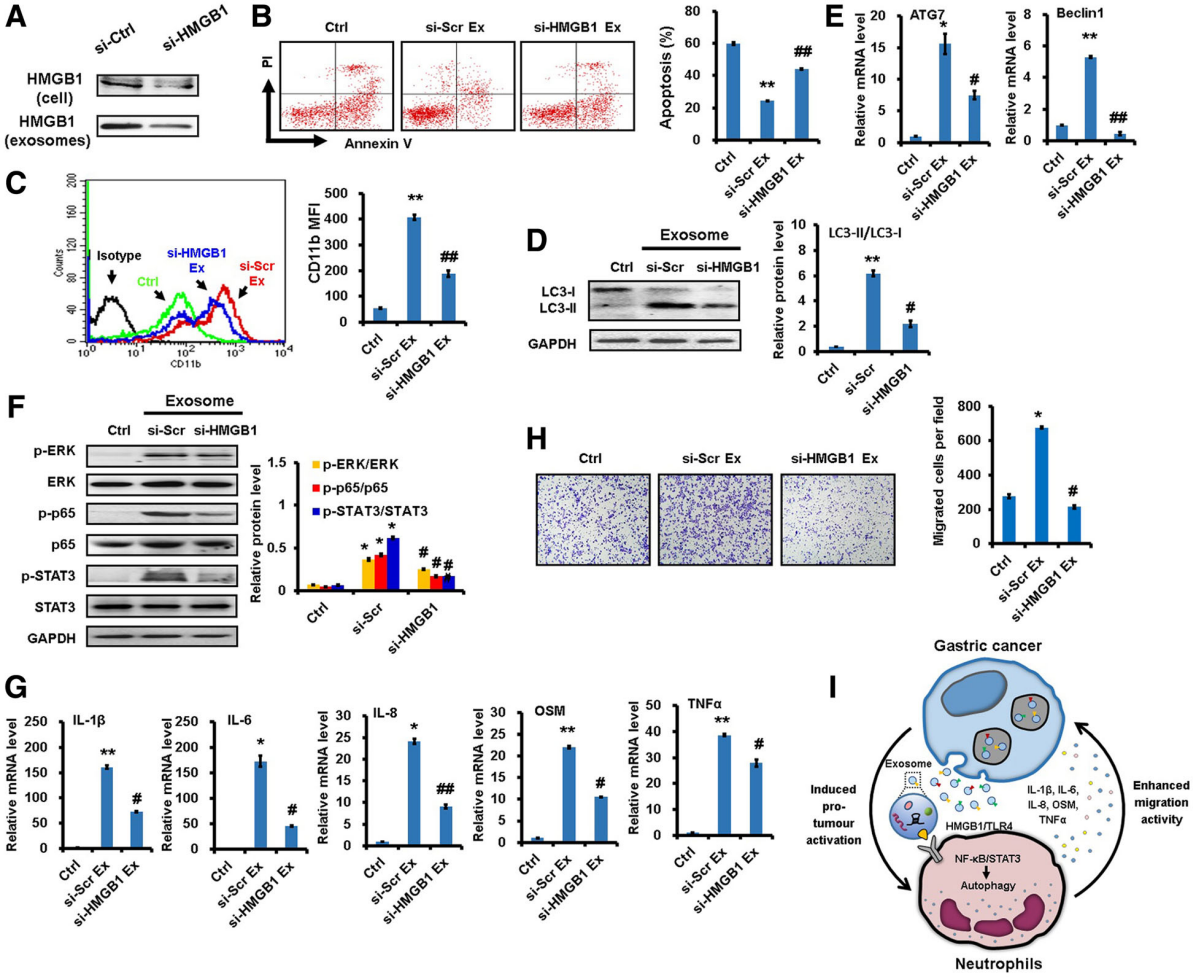
HMGB1 knockdown impaired neutrophil activation by gastric cancer cell-derived exosomes. *HMGB1* was silenced in gastric cancer cell by siRNA. Neutrophils were treated with exosomes from *HMGB1*-silenced (si-*HMGB1*) and scramble control siRNA (si-Scr) transfected gastric cancer cells. **a**. Western blot assays for HMGB1 expression in gastric cancer cells transfected with HMGB1 siRNA and their derived exosomes. **b-c**. The percentage of apoptotic cells (**b**) and CD11b expression (**c**) in neutrophils were determined by FACS analyses. **d**. Western blot assays for LC3-II expression in neutrophils. **e**. The expression of *ATG7* and *BECN1* genes is measured by qRT-PCR. **f**. The expression of p-p65, p-STAT3 and p-ERK in neutrophils was determined by western blot. **g**. The expression of inflammatory factors in neutrophils was measured by qRT-PCR. **h**. Gastric cancer cells was incubated with the supernatants from neutrophils and subjected to transwell migration assay. **i**. Proposed model for the role of tumor-derived exosomes in the pro-tumor activation of neutrophils to promote gastric cancer cell migration. ^**^P<0.01 and ^*^P<0.05 compared to control (Ctrl); ^##^*P*<0.01 and ^#^*P*<0.05 compared to si-Scr Ex

To clarify the importance of HMGB1 in the pro-tumor activation of neutrophils, we silenced *HMGB1* in gastric cancer cells by gene-specific siRNA (Fig. 7a) and tested GC-Ex-induced neutrophil activation. Compared to exosomes from cells transfected with scramble control siRNA, exosomes from *HMGB1*-silenced cells showed decreased protection against spontaneous apoptosis and CD11b expression in neutrophils (Fig. 7b and c). In addition, *HMGB1* knockdown decreased GC-Ex-induced LC3-II expression as well as *ATG7* and *BECN1* expression in neutrophils (Fig. 7d and e). Similarly, GC-Ex from *HMGB1*-silenced cells had decreased ability to activate NF-κB pathway in neutrophils compared to control exosomes (Fig. 7f). Furthermore, inflammatory factor expression decreased for neutrophils that were treated with exosomes from *HMGB1*-silenced cancer cells compared to exosomes from cells transfected with scramble control siRNA (Fig. 7g). Finally, HMGB1 knockdown blocked the promoting effect of GC-Ex-activated neutrophils on gastric cancer cell migration (Fig. 7h). On the contrary, recombinant HMGB1 inhibited spontaneous apoptosis and increased CD11b expression in neutrophils (Additional file 9: Figure S8A and B). Recombinant HMGB1-treated neutrophils promoted the migration of gastric cancer cells and increased the expression of *ATG7* and *BECN1* genes in neutrophils (Additional file 9: Figure S8C and D). The expression of MMP9 and VEGF and pro-inflammatory factors was also upregulated in recombinant HMGB1-treated neutrophils (Additional file 9: Figure S8E). These results support HMGB1 as a key mediator for GC-Ex-induced neutrophil activation.

## Discussion

In this study, we reported that gastric cancer cells induced a pro-tumor phenotype in neutrophils via exosomes. Gastric cancer cell-derived exosomes induced autophagy in neutrophils by activating NF-κB pathway through HMGB1/TLR4 interaction. Activated neutrophils, in turn, promoted gastric cancer cell migration *in vitro*. Our findings reveal a novel mechanism for neutrophil modulation in the tumor milieu and provide new evidence for the important roles of exosomes in tumor microenvironment.

Inflammation in tumor microenvironment is a hallmark of cancer. Immune cells can be modulated by tumor signals to acquire tumor-promoting phenotype. The previous studies have shown that neutrophils could be redirected to a pro-tumor phenotype at the late stage of tumor progression [11]. The presence of neutrophils in tumors is considered as an independent and unfavorable factor for the prognosis of gastric cancer patients [30]. Wu *et al*. demonstrated that the number of neutrophils infiltrated in gastric cancer tissues was positively associated with lymph node metastasis. Furthermore, the supernatant from gastric cancer cells induced IL-1β and TNFα expression in neutrophils and prolonged the half-life of neutrophils [18]. In consistent with this report, we found that gastric cancer cell-derived conditioned medium protected neutrophils from spontaneous apoptosis and induced IL-1β and TNFα expression, among other inflammatory factors. Although tumor-derived HA (hyaluronan) fragments can mediate neutrophil activation [18, 31], we showed in the current study that gastric cancer cell-derived exosomes could induce neutrophil activation, suggesting that both soluble factors and non-soluble extracellular vehicles produced by tumor cells could induce neutrophil activation. Moreover, neutrophils activated by gastric cancer cell-derived exosomes highly express several inflammatory factors (such as IL-1β and OSM) that have been previously shown to promote cancer cell migration and invasion [18, 32], suggesting that the activated neutrophils may promote gastric cancer metastasis by releasing these factors.

Autophagy, a mechanism for intracellular degradation and energy recycling, is emerging as an important regulator of immune responses. Autophagy has been linked to the generation, expansion, and function of neutrophils. Autophagy deficiency reduces degranulation in neutrophils, suggesting the requirement of autophagy for neutrophil-mediated inflammation [33]. In addition, autophagy is essential for intracellular bacterial killing by human neutrophils [34]. Kimmey *et al*. suggest that stimulating autophagy in neutrophils increases bacterial killing but inhibiting autophagy increases bacterial survival [35], indicating that autophagy is essential for the function of neutrophils. It has been reported that granulocyte-colony stimulating factor (G-CSF) activates autophagy in neutrophils and G-CSF-induced neutrophil expansion is impaired in the absence of autophagy [36]. More recently, Li *et al*. suggest that increased autophagy sustains the pro-tumor effects of neutrophils in human hepatocellular carcinoma [31]. Interestingly, Dutta *et al.* demonstrate that exosomes from breast cancer cells induce autophagy in primary mammary epithelial cells, which in turn, produce factors to promote breast cancer cell growth [37]. We found that exosomes from gastric cancer cells induced autophagy in neutrophils, leading to the release of factors that promoted the migration ability of gastric cancer cells. Thus, our results, together with findings from the others, indicate that tumor-derived exosomes may regulate neutrophil phenotype and function by inducing autophagic activation.

Interaction of HMGB1 and TLR4 is involved in infection, tissue injury, and cancer [38]. The previous studies have shown that UV irradiation-damaged skin cells produce HMGB1 that recruits and activates neutrophils, promoting angiogenesis and melanoma metastasis [39]. In addition, tumor cell-derived HMGB1 mediates tumor cell-platelet interaction to promote metastasis [40]. HMGB1 is shown to be generated in a vesicle form in monocytes and tumor cells [40, 41]. Activated platelets present HMGB1 to neutrophils, inducing autophagy and promoting the formation of neutrophil extracellular traps [42]. In this study, we found that HMGB1 was present in GC-Ex. Inhibition of HMGB1/TLR4 interaction suppressed GC-Ex-induced pro-tumor activation of neutrophils, supporting that HMGB1 is a key factor for the roles of GC-Ex. However, this does not necessarily exclude other factors in GC-Ex that may contribute to neutrophil activation. Finally, overexpression of HMGB1 is reported to be associated with adverse prognosis in cancer patients [43]. Indeed, we also found that increased HMGB1 expression in gastric cancer patients was associated with poor outcomes. Nonetheless, large cohort studies are warranted to determine the potential of exosomal HMGB1 as a biomarker for gastric cancer diagnosis and prognosis.

Tumor-derived exosomes can promote tumor growth by regulating immune cell phenotype and function [44]. For instance, tumor-derived exosomes act on T cells [45, 46], NK cells [12, 47], macrophages [48, 49], dendritic cells [50], and myeloid-derived suppressor cells (MDSCs) [51] to induce an immunosuppressive microenvironment. Exosomes from ovarian cancer cells polarize macrophages to an M2 phenotype that, in turn, promotes ovarian cancer growth and metastasis [52]. Similarly, neutrophils can be polarized to an N2 phenotype by tumor-derived factors in murine tumor models [53]. The N2-polarized neutrophils display pro-metastatic and immunosuppressive activities [53–55]. Although polarization of neutrophils in human cancers has not been well characterized, we showed in this study that exosomes from human gastric cancer cells induced neutrophils to represent an N2-like phenotype, supporting a role of tumor-derived exosomes in neutrophil polarization.

Neutrophils in the circulation can be divided into high density neutrophils (HDN) and low density neutrophils (LDN). In various diseases including cancer, the frequency of LDN increased [56]. Sagiv *et al*. suggest that LDN can be induced from HDN by TGF-β stimulation to promote cancer progression [56]. It should be noted that in this study, HDN were isolated whereas low density neutrophils (LDN) were excluded due to the density gradient preparation method we used. The effects of tumor derived exosomes on the phenotype and function of LDN will be tested in future studies.

## Conclusion

In conclusion, we demonstrate in this study that gastric cancer cell-derived exosomes induce autophagy and pro-tumor activation in neutrophils through the HMGB1/TLR4/NF-кB signaling pathway, which finally promotes the proliferation and migration of gastric cancer cells (Fig. 7i). These findings provide new insight into the mechanisms by which neutrophils are regulated in tumor microenvironment and contribute to tumor growth and metastasis, and will permit the development of new strategies for gastric cancer diagnosis, prognosis, and therapy.

## Methods

### Patients and biopsy specimens

Fresh tissue specimens were obtained from patients with GC who underwent surgical resection at the Affiliated People’s Hospital of Jiangsu University between April 2016 and December 2016. None of these patients had received chemotherapy or radiotherapy before surgery. Patients with infectious diseases, autoimmune disease or multi-primary cancers were excluded. The study was approved by the ethics committee of Jiangsu University and informed consent was obtained from all patients.

### Cell culture

Human gastric cancer cell lines BGC-823, MGC80-3, SGC-7901, and HGC-27 were purchased from the Institute of Biochemistry and Cell Biology at the Chinese Academy of Sciences (Shanghai, China). Cells were cultured in low-glucose Dulbecco's modified Eagle's medium (DMEM), supplemented with 10% fetal bovine serum (FBS; Invitrogen, Carlsbad, CA, USA) at 37 ^o^C in humidified air with 5% CO_2_. When cells reached 80% confluence, medium was replaced with serum-free medium. Following a 24-hour incubation, medium was collected, centrifuged to remove cell debris, and stored at -80 ^o^C in aliquots as conditioned medium (CM).

### Neutrophil isolation and treatment

Peripheral blood was collected from healthy volunteers after obtaining written informed consent. The study was approved by the ethics committee of Jiangsu University. Neutrophils were isolated by using Polymorphprep (Axis-Shield PoC AS, Norway) as previously described [21]. RBCs were lysed using hypotonic lysing procedure. The purity of neutrophils was 98% after this procedure. Neutrophils were seeded in RPMI 1640 (Invitrogen) supplemented with 10% FBS and 1% penicillin/streptomycin at a density of 1×10^6^ per well and treated with or without CM or exosomes from gastric cancer cells for 12 h. For experiments that use inhibitors, cells were pretreated with inhibitors for 1 h prior to adding CM or exosomes. The inhibitors used in this study include autophagy initiation inhibitor 3-methyladenine (3-MA, 5 mM), autophagosome degradation inhibitor chloroquine (CQ, 20 μM), HMGB1 inhibitor Glycyrrhizin (Gly, 10 μM), TLR4 antagonist TAK-242 (TAK, 10 μM), ERK inhibitor U0126 (10 μM), NF-κB inhibitor Bay11-7082 (10 μM), and STAT3 inhibitor WP1066 (10 μM). For HMGB1 treatment, cells were treated with recombinant human HMGB1 (10 μg/ml; Biovision, Shanghai, China) for 12 hours.

### Exosome isolation

Exosomes were isolated from the conditioned medium of gastric cancer cells as previously described [29]. In brief, cells were cultured in exosome-depleted medium and the supernatants were collected after 48 h. The conditioned medium was centrifuged at 1,000 *g* for 10 min to remove cell debris followed by 30 min at 10,000 *g* using 100 KDa MWCO before the concentrated solutions were filtrated through a 0.22-μm pore filter (Millipore, Shanghai, China). Exosomes were precipitated by adding the exosome quick extraction solution (System Biosciences, Palo Alto, CA, USA) at a ratio of 1:5 at 4 ^o^C for 12 h. Exosomes were dissolved with PBS and stored at -80 ^o^C. Protein concentration was determined by BCA protein assay kit (ThermoFisher Scientific, Shanghai, China). The size and concentration of exosomes were measured by Nanoparticle Tracking Analysis (NTA). The morphology of purified exosomes was identified by transmission electron microscopy (Tecnai 12; Philips) and the expression of exosomal markers CD9 and CD81 by western blot.

### LC-MS/MS

Exosome samples (250 μg) were lysed in STD buffer (4% SDS, 100 mM Tris/Hcl, and 1 mM dithiothreitol, pH 7.6) and centrifugated at 1000 *g* for 10 min to collect the supernatants. Proteins were identified using Q Exactive Orbitrap LC-MS/MS system (ThermoFisher Scientific).

### Tissue microarray

Tissue array was purchased from Shines Pharmaceuticals (Shanghai, China). A total of 76 pairs of tumor tissues and non-tumor tissues were included in the tissue array. Tissue array was incubated with antibody against HMGB1 (Cell Signaling Technology). Immunohistochemical staining was performed as described elsewhere. IHC scoring was assessed by two pathologists in a double-blinded manner.

### ELISA

The supernatants from gastric cancer tissues and adjacent non-cancerous tissues were collected for ELISA. The concentrations of HMGB1 in tissue supernatants were determined by using ELISA kit according to the manufacturer’s instructions (Chondrex, Redmond, WA, USA).

### ROS detection assay

Neutrophils treated with or without CM or exosome from gastric cancer cells for 12 h were collected and resuspended in serum-free medium. Cells were stained with DCFH-DA (10 μM; Beyotime Biotechnology, Shanghai, China) at 37 ^o^C for 30 min and subjected to analyses of florescence intensity by flow cytometry.

### Real-time quantitative PCR

Total RNA was extracted from cells using Trizol reagent (Thermo Fisher Scientific) and 1 μg of RNA was reverse transcribed to cDNA by using reverse transcriptase (Vazyme). Real-time quantitative PCR was performed by using the SYBR Green I real-time detection kit (Cwbio, Beijing, China) on a Bio-Rad CFX96 Detection System. The relative gene expression was normalized to β-actin. The primers for target genes were listed in Additional file 10: Table S1.

### RNA interference

The siRNA against HMGB1 was produced by Genepharrm (Suzhou, Jiangsu, China). The sequences of HMGB1 siRNA and the scramble control were shown in Additional file 11: Table S2. BGC-823 cells (1×10^5^ cells/well) were grown in 6-well plates and transfected with siRNAs by using LipoFiter transfection reagent (Hanbio, Shanghai, China) for 36 hours.

### Western blot

Cells were lysed in RIPA buffer containing proteinase inhibitors. Equal amount of proteins was separated by a 12% SDS-PAGE gel. Following electrophoresis, proteins were transferred to a PVDF membrane, blocked in 5% non-fat milk, and incubated with primary antibodies at 4 ^o^C overnight. Antibodies for ERK1/2, p-ERK1/2, NF-кB p65, p-p65, STAT3, p-STAT3, p-p38, p38, p-Akt, Akt, LC3, CD9, CD63, Alix, and TSG101 were purchased from Cell Signaling Technology (Louis Park, MN, USA). After washing with TBST for three times, membrane was incubated with HRP-conjugated goat anti-rabbit or anti-mouse secondary antibodies (Bioworld Technology) at room temperature for two hours. The protein bands were visualized by enhanced chemiluminescence. GAPDH served as the loading control.

### Cell apoptosis assay

Neutrophil apoptosis was analyzed by using an Annexin V apoptosis detection kit according to the manufacturer’s instructions (Invitrogen). The binding of Annexin V-FITC and PI to the cells was analyzed on FACS Calibur (BD Biosciences, NJ, USA) by using Cell Quest software.

### Autophagosome detection

For transmission electron microscopic analysis, neutrophils treated with or without CM or exosome from gastric cancer cells were washed and fixed in 4% glutaraldehyde, followed by post-fixation in 2% osmium tetroxide. Thereafter, cells were dehydrated, treated with propylene oxide, and embedded. The sections were subsequently stained with uranyl acetate and lead citrate and examined in a Tecnai 12 transmission electron microscopy. For immunofluorescent staining, neutrophils treated with or without CM or exosomes were stained with an autophagy detection kit (Enzo Lifesciences, NY, USA) and analyzed by Cytasion 3 cell imaging multi-mode reader (BioTek, Shanghai, China).

### Transwell migration assay

Cell migration assay was performed in a 24-well Boyden chamber with an 8-μm pore size polycarbonate membrane (Corning, Union City, CA, USA). Cancer cells (2×10^4^ in 100 μl of serum-free medium) were added into the upper chamber with 600 μl of supernatant from neutrophils in the lower chamber. After a 12-h incubation, cancer cells on the upper surface of the membrane were removed. The migrated cells on the lower surface of the membrane were fixed by paraformaldehyde, stained with crystal violet, and counted under a microscope.

### Tube formation assay

Neutrophils were treated with or without CM or exosome from gastric cancer cells for 12 h, followed by washing with PBS once and culturing in fresh medium for additional 12 h. The supernatant from activated neutrophils were collected and filtered through a 0.22 μm filter. Human umbilical vein endothelial cells (HUVEC) were seeded at 2×10^4^ cells/well and incubated with or without conditioned media from neutrophils at 37 ^o^C for 12 h. The formation of tube-like structure by HUVECs were observed under a phase-contrast microscope and photographed at 100× magnification. The number of tubules from five random fields in each well was counted. The experiments were repeated for three times.

### CCK8 assay

Gastric cancer cells were seeded at 4×10^3^ cells/well and incubated with or without supernatant from the activated neutrophils. CCK8 reagent (10 μl; Vazyme, Nanjing, China) was added at 3 hours before the end of the experiments. The absorption was measured at 450nm in a microplate reader. The experiments were repeated for three times.

### Tumor tissue-derived conditioned medium and exosomes

Fresh surgically removed gastric cancer tissues and adjacent non-cancerous tissues (at least 5 cm distant from the tumor site) were cut into 1 cm^3^ and placed in 1 ml serum-free RPMI 1640 medium for 24 h. The supernatants were centrifuged at 300 *g* for 10 min and filtered through a 0.22 μm filter and stored at -80 ^o^C until use. Neutrophils were cultured in 50% T-CM or N-CM for 12 h. Tumor-derived exosomes were extracted from the conditioned medium as described in previous section.

### Statistical analysis

Data were expressed as means ± SD from at least three independent experiments. The statistical significance of differences between groups was determined by two-tailed Student’s t test. Survival time was analyzed by Kaplan–Meier method and log-rank test. *P*<0.05 was considered statistically significant.

## Additional files

Additional file 1: **Figure S1.** Gastric cancer cell-derived conditioned medium induced pro-inflammatory factor gene expression in neutrophils. A. QRT-PCR analyses of pro-inflammatory factor gene expression (IL-1β, IL-6, IL-8, OSM, and TNFα) in BGC-CM-treated neutrophils. B. The expression of MMP-9, VEGF, CXCR2, and TLR4 in BGC-CM-treated neutrophils was determined by qRT-PCR. ^**^P<0.01, and ^*^P<0.05 compared to control. (JPG 947 kb)

Additional file 2: **Figure S2.** BGC-CM-treated neutrophils promoted gastric cancer cell proliferation and endothelial cell tube formation. A. ROS production in BGC-CM-treated neutrophils was measured by flow cytometric analysis. B. Wright’s stain for BGC-CM-treated neutrophils. Magnification, 200×. C. The proliferation rate of gastric cancer cells treated with supernatant from BGC-CM-treated neutrophils was measured by CCK8 assay. D. Tube formation assay for endothelial cells following treatment with supernatant from BGC-CM-treated neutrophils. E. Western blot assays for p38 and Akt expression in neutrophils treated with BGC-CM. ^**^P<0.01, and ^*^P<0.05 compared to control. (JPG 1917 kb)

Additional file 3: **Figure S3.** NF-κB inhibitor blocked BGC-CM-induced pro-inflammatory factor gene expression in neutrophils. Neutrophils were pre-treated with NF-κB, STAT3 or ERK inhibitors followed by incubation with gastric cancer cell-derived conditioned medium. The expression of pro-inflammatory factor genes was determined by qRT-PCR. ^**^P<0.01 and ^*^P<0.05 compared to control; ^##^*P*<0.01 and ^#^*P*<0.05 compared to BGC-CM. (JPG 1511 kb)

Additional file 4: **Figure S4.** BGC-Ex-treated neutrophils promoted gastric cancer cell proliferation and endothelial cell tube formation. A. ROS production in BGC-Ex-treated neutrophils was measured by flow cytometric analysis. B. Wright’s stain for BGC-Ex-treated neutrophils. Magnification, 200×. C. The proliferation rate of gastric cancer cells treated with supernatant from BGC-Ex-treated neutrophils was measured by CCK8 assay. D. T Tube formation assay for endothelial cells following treatment with supernatant from BGC-Ex-treated neutrophils. E. qRT-PCR assays for MMP-9, VEGF, CXCR2, and TLR4 expression in BGC-Ex-treated neutrophils. F. Western blot assays for p38 and Akt expression in neutrophils treated with BGC-Ex. ^**^P<0.01, and ^*^P<0.05 compared to control. (JPG 2483 kb)

Additional file 5: **Figure S5.** Gastric cancer cell-derived exosomal proteins induced autophagy and promoted the activation of neutrophils. A. Western blot assays for the expression of LC3-II in neutrophils treated with undigested or proteinase-digested BGC-Ex. B. The expression of *ATG7* and *BECN1* genes in neutrophils treated with undigested or proteinase-digested BGC-Ex was measured by qRT-PCR. C. Western blot assays for NF-κB, STAT3, and ERK expression in neutrophils treated with undigested or proteinase-digested BGC-Ex. D. QRT-PCR analyses for pro-inflammatory factor expression in neutrophils treated with undigested or proteinase-digested BGC-Ex. E. Neutrophils were treated with BGC-Ex in the presence of TLR4 neutralizing antibody. The expression of pro-inflammatory factor genes was determined by qRT-PCR. ^**^P<0.01 and ^*^P<0.05 compared to control; ^##^*P*<0.01 and ^#^*P*<0.05 compared to BGC-Ex. (JPG 1486 kb)

Additional file 6: **Figure S6.** Autophagy is a common mechanism for the activation of neutrophils induced by gastric cancer cell-derived exosomes. A. Flow cytometric analyses of the percentage of apoptotic neutrophils following treatment with gastric cancer cell-derived exosomes. B. FACS analyses of CD11b expression in neutrophils treated with gastric cancer cell-derived exosomes. C. The expression of LC3-II in gastric cancer cell-derived exosomes-treated neutrophils was detected by western blot. D. QRT-PCR analyses of *ATG7* and *BECN1* gene expression in gastric cancer cell-derived exosomes-treated neutrophils. E. The expression of pro-inflammatory factors (IL-1β, IL-6, IL-8, OSM, and TNFα) in gastric cancer cell-derived exosomes-treated neutrophils was determined by qRT-PCR. F. Transwell migration assays for gastric cancer cells after treatment with supernatant from gastric cancer cell-derived exosomes-treated neutrophils. ^**^P<0.01 and ^*^P<0.05 compared to control (Ctrl). (JPG 2357 kb)

Additional file 7: **Table S3.** The list of proteins identified in all the gastric cancer cells derived exosomes. (DOCX 18 kb)

Additional file 8: **Figure S7.** Exosomal HMGB1 expression in tumor tissues and serum samples of gastric cancer patients. A. The expression of exosomal HMGB1 in the culture supernatants of tumor tissues and adjacent normal tissues (n=3) of gastric cancer patients was detected by using western blot. B. The expression of exosomal HMGB1 in the serum samples of healthy controls and gastric cancer patients (n=3) was detected by using western blot. (JPG 996 kb)

Additional file 9: **Figure S8.** Recombinant HMGB1 induced autophagy and promoted the activation of neutrophils. A. The percentage of apoptotic neutrophils following treatment with recombinant HMGB1 was determined by flow cytometric analyses. B. Flow cytometric analyses of CD11b expression in recombinant HMGB1-treated neutrophils. C. Transwell migration assays for gastric cancer cells after treatment with supernatant from recombinant HMGB1-treated neutrophils. D. The expression of *ATG7* and *BECN1* genes in recombinant HMGB1-treated neutrophils was measured by qRT-PCR. E. The expression of MMP-9, VEGF, CXCR2, and TLR4 genes in neutrophils treated with recombinant HMGB1 was determined by qRT-PCR. F. The expression of pro-inflammatory factors (IL-1β, IL-6, IL-8, OSM, and TNFα) in neutrophils treated with recombinant HMGB1 was measured by qRT-PCR. ^***^P<0.001, ^**^P<0.01, and ^*^P<0.05 compared to control. (JPG 1651 kb)

Additional file 10: **Table S1.** The sequences of primers for qPCR. (DOCX 18 kb)

Additional file 11: **Table S2.** The target sequence of HMGB1 siRNA. (DOCX 15 kb)
