# Supplementary figures and images for: Tumor-derived exosomes induce N2 polarization of neutrophils to promote gastric cancer cell migration
Source: Mol Cancer. 2018 Oct 6;17:146. doi: 10.1186/s12943-018-0898-6 (PMC6174070; doi:10.1186/s12943-018-0898-6)

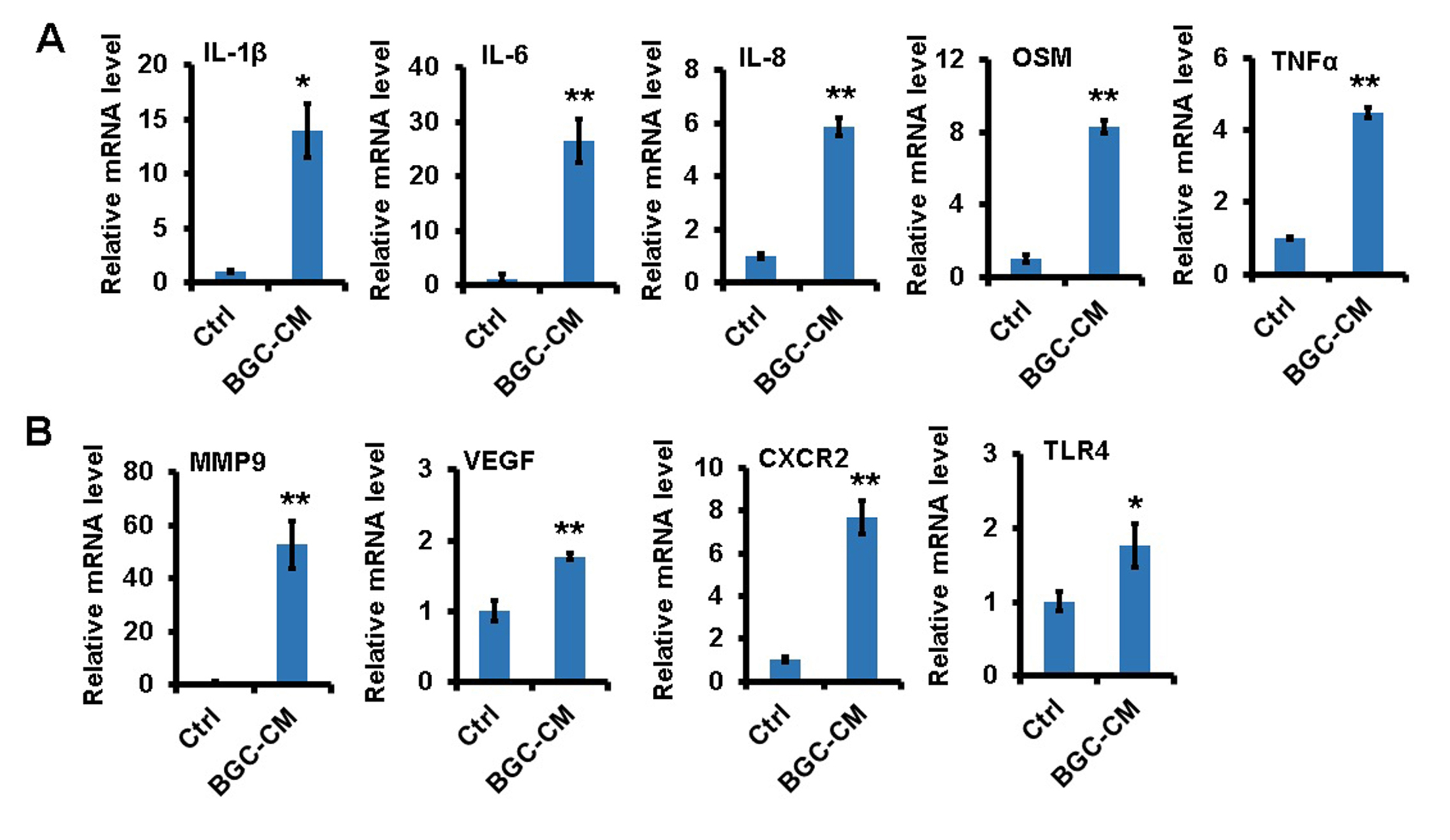

Supplement: Supplementary file 1 — Figure S1. Gastric cancer cell-derived conditioned medium induced pro-inflammatory factor gene expression in neutrophils. A. QRT-PCR analyses of pro-inflammatory factor gene expression (IL-1β, IL-6, IL-8, OSM, and TNFα) in BGC-CM-treated neutrophils. B. The expression of MMP-9, VEGF, CXCR2, and TLR4 in BGC-CM-treated neutrophils was determined by qRT-PCR. **P<0.01, and *P<0.05 compared to control. (JPG 947 kb) [file 12943_2018_898_MOESM1_ESM.jpg]

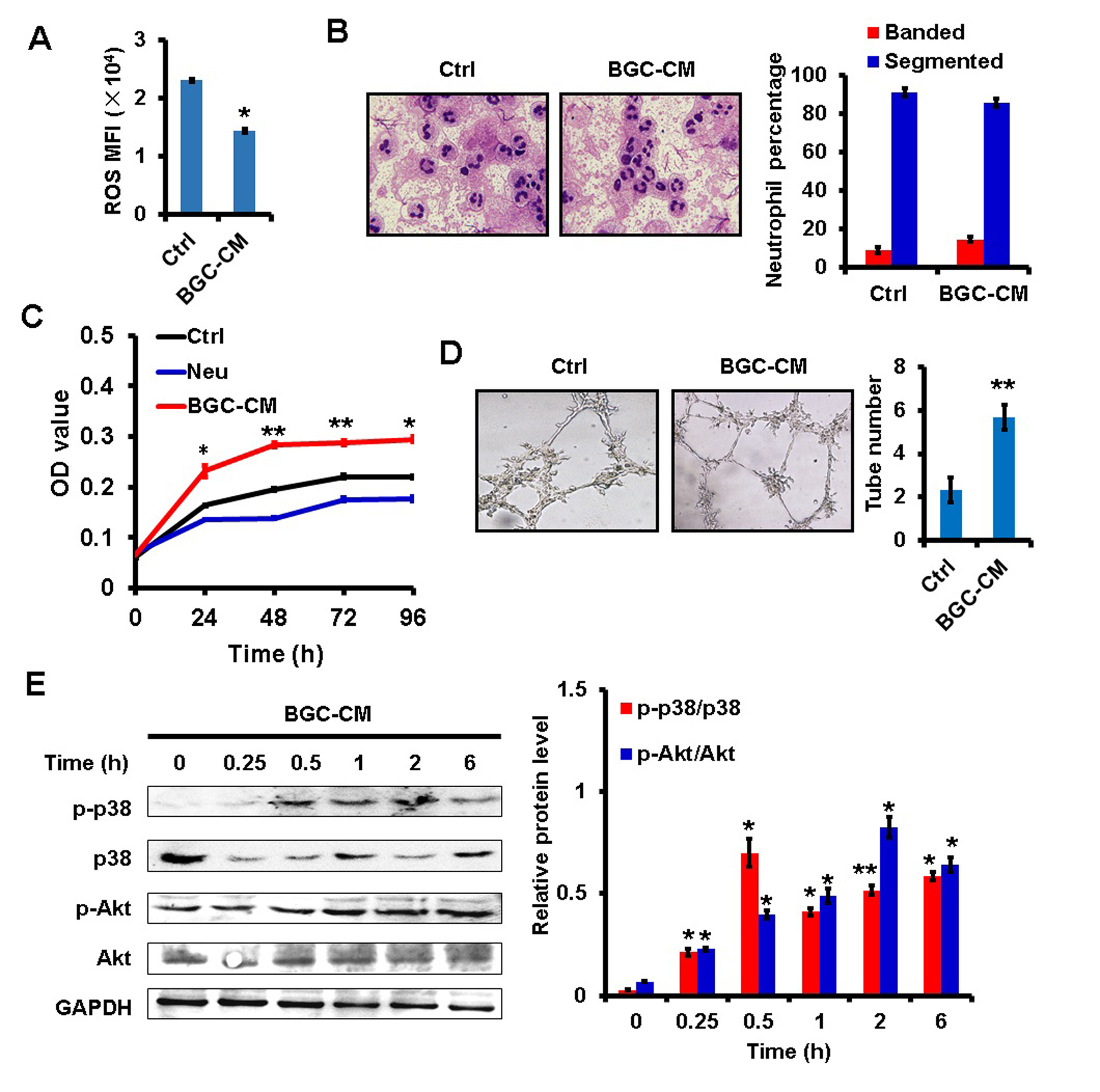

Supplement: Supplementary file 2 — Figure S2. BGC-CM-treated neutrophils promoted gastric cancer cell proliferation and endothelial cell tube formation. A. ROS production in BGC-CM-treated neutrophils was measured by flow cytometric analysis. B. Wright’s stain for BGC-CM-treated neutrophils. Magnification, 200×. C. The proliferation rate of gastric cancer cells treated with supernatant from BGC-CM-treated neutrophils was measured by CCK8 assay. D. Tube formation assay for endothelial cells following treatment with supernatant from BGC-CM-treated neutrophils. E. Western blot assays for p38 and Akt expression in neutrophils treated with BGC-CM. **P<0.01, and *P<0.05 compared to control. (JPG 1917 kb) [file 12943_2018_898_MOESM2_ESM.jpg]

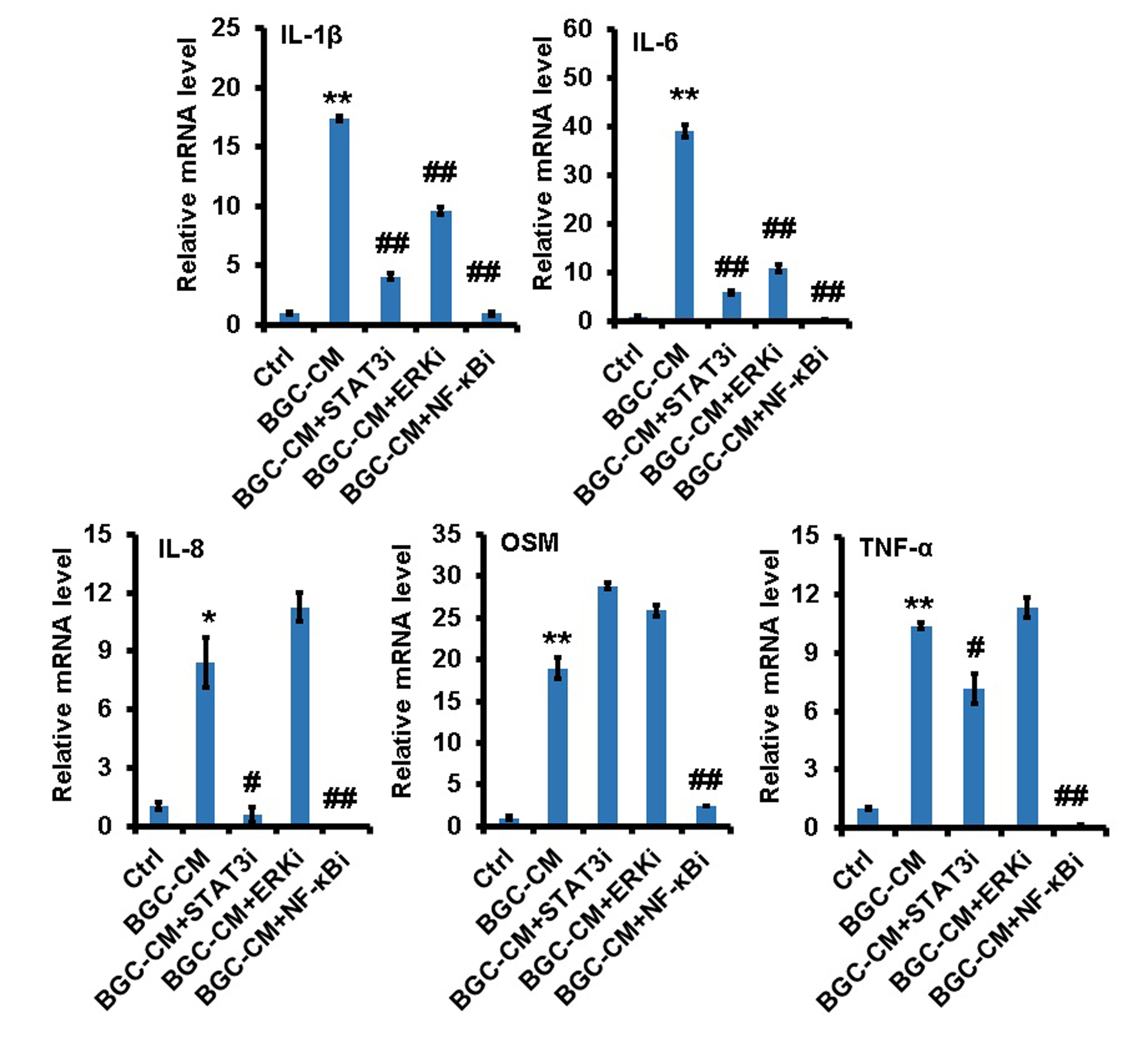

Supplement: Supplementary file 3 — Figure S3. NF-κB inhibitor blocked BGC-CM-induced pro-inflammatory factor gene expression in neutrophils. Neutrophils were pre-treated with NF-κB, STAT3 or ERK inhibitors followed by incubation with gastric cancer cell-derived conditioned medium. The expression of pro-inflammatory factor genes was determined by qRT-PCR. **P<0.01 and *P<0.05 compared to control; ##P<0.01 and #P<0.05 compared to BGC-CM. (JPG 1511 kb) [file 12943_2018_898_MOESM3_ESM.jpg]

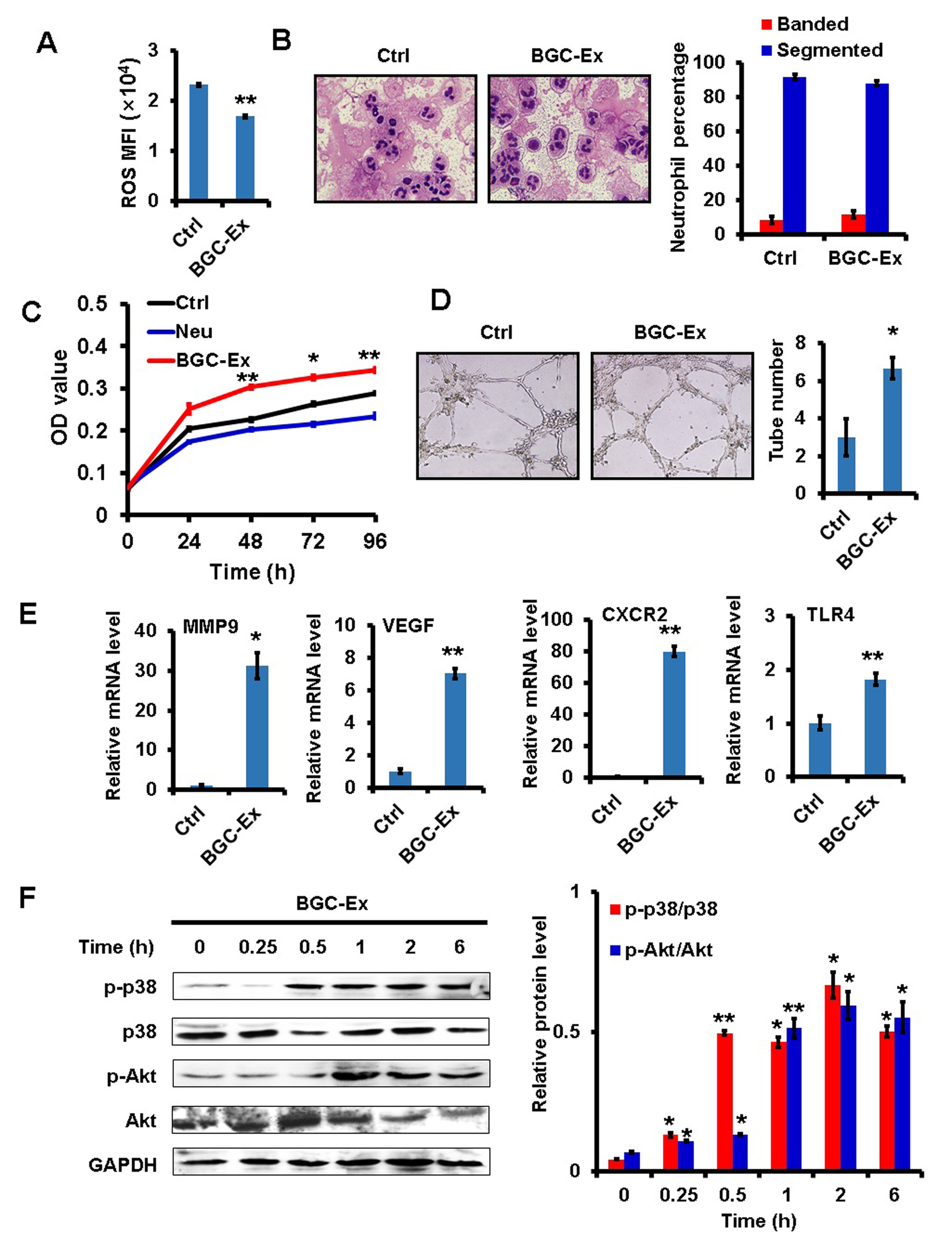

Supplement: Supplementary file 4 — Figure S4. BGC-Ex-treated neutrophils promoted gastric cancer cell proliferation and endothelial cell tube formation. A. ROS production in BGC-Ex-treated neutrophils was measured by flow cytometric analysis. B. Wright’s stain for BGC-Ex-treated neutrophils. Magnification, 200×. C. The proliferation rate of gastric cancer cells treated with supernatant from BGC-Ex-treated neutrophils was measured by CCK8 assay. D. T Tube formation assay for endothelial cells following treatment with supernatant from BGC-Ex-treated neutrophils. E. qRT-PCR assays for MMP-9, VEGF, CXCR2, and TLR4 expression in BGC-Ex-treated neutrophils. F. Western blot assays for p38 and Akt expression in neutrophils treated with BGC-Ex. **P<0.01, and *P<0.05 compared to control. (JPG 2483 kb) [file 12943_2018_898_MOESM4_ESM.jpg]

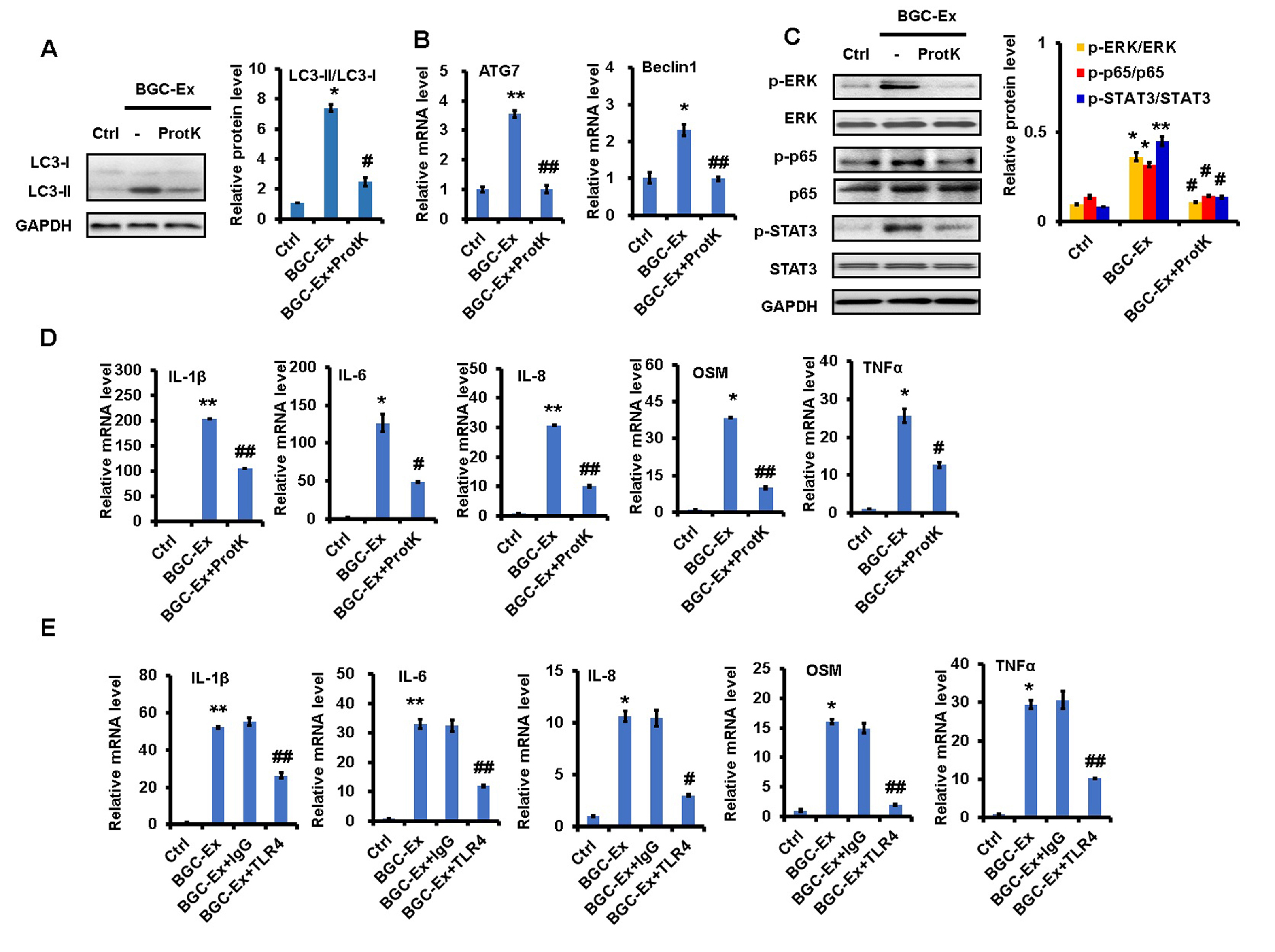

Supplement: Supplementary file 5 — Figure S5. Gastric cancer cell-derived exosomal proteins induced autophagy and promoted the activation of neutrophils. A. Western blot assays for the expression of LC3-II in neutrophils treated with undigested or proteinase-digested BGC-Ex. B. The expression of ATG7 and BECN1 genes in neutrophils treated with undigested or proteinase-digested BGC-Ex was measured by qRT-PCR. C. Western blot assays for NF-κB, STAT3, and ERK expression in neutrophils treated with undigested or proteinase-digested BGC-Ex. D. QRT-PCR analyses for pro-inflammatory factor expression in neutrophils treated with undigested or proteinase-digested BGC-Ex. E. Neutrophils were treated with BGC-Ex in the presence of TLR4 neutralizing antibody. The expression of pro-inflammatory factor genes was determined by qRT-PCR. **P<0.01 and *P<0.05 compared to control; ##P<0.01 and #P<0.05 compared to BGC-Ex. (JPG 1486 kb) [file 12943_2018_898_MOESM5_ESM.jpg]

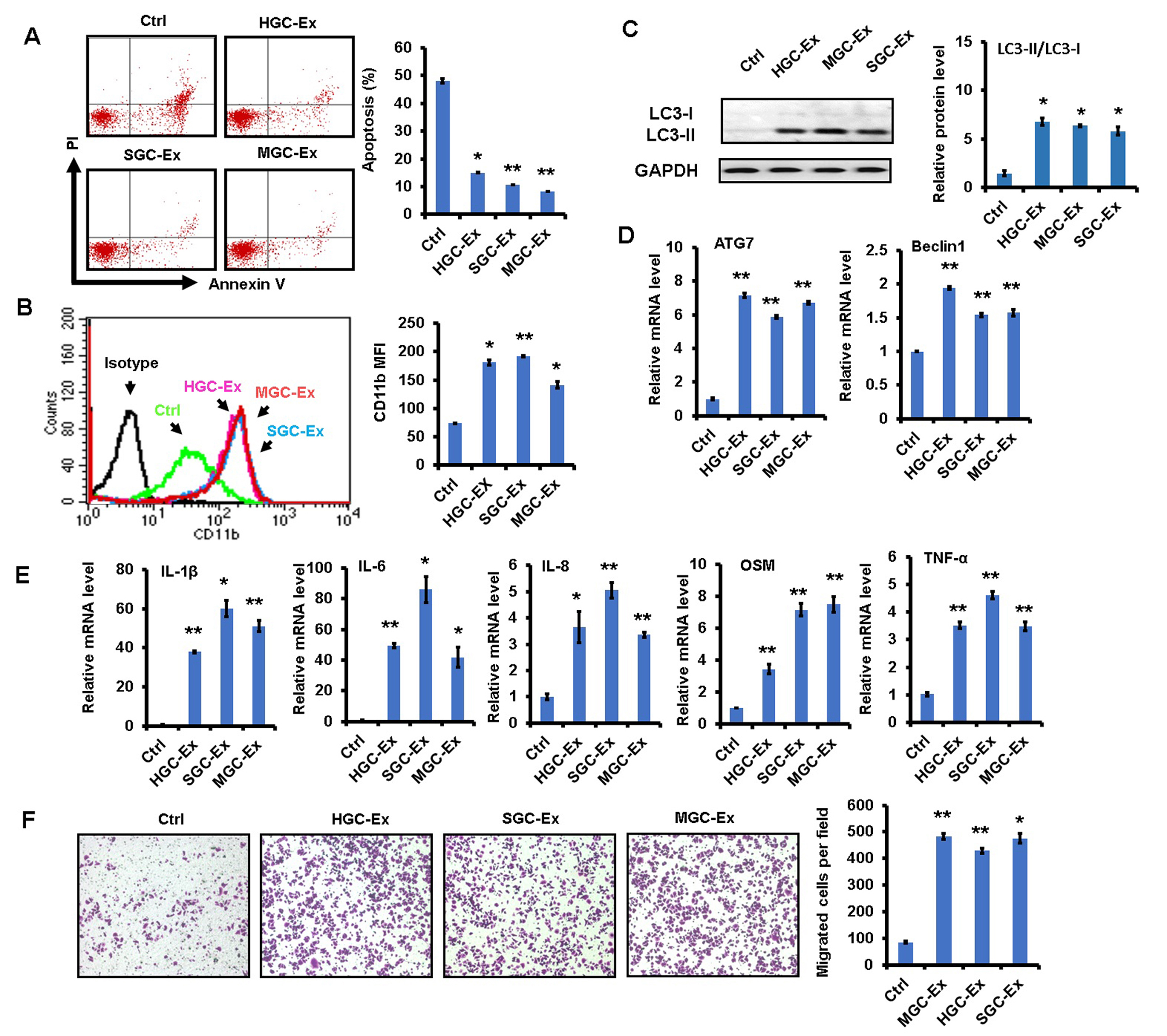

Supplement: Supplementary file 6 — Figure S6. Autophagy is a common mechanism for the activation of neutrophils induced by gastric cancer cell-derived exosomes. A. Flow cytometric analyses of the percentage of apoptotic neutrophils following treatment with gastric cancer cell-derived exosomes. B. FACS analyses of CD11b expression in neutrophils treated with gastric cancer cell-derived exosomes. C. The expression of LC3-II in gastric cancer cell-derived exosomes-treated neutrophils was detected by western blot. D. QRT-PCR analyses of ATG7 and BECN1 gene expression in gastric cancer cell-derived exosomes-treated neutrophils. E. The expression of pro-inflammatory factors (IL-1β, IL-6, IL-8, OSM, and TNFα) in gastric cancer cell-derived exosomes-treated neutrophils was determined by qRT-PCR. F. Transwell migration assays for gastric cancer cells after treatment with supernatant from gastric cancer cell-derived exosomes-treated neutrophils. **P<0.01 and *P<0.05 compared to control (Ctrl). (JPG 2357 kb) [file 12943_2018_898_MOESM6_ESM.jpg]

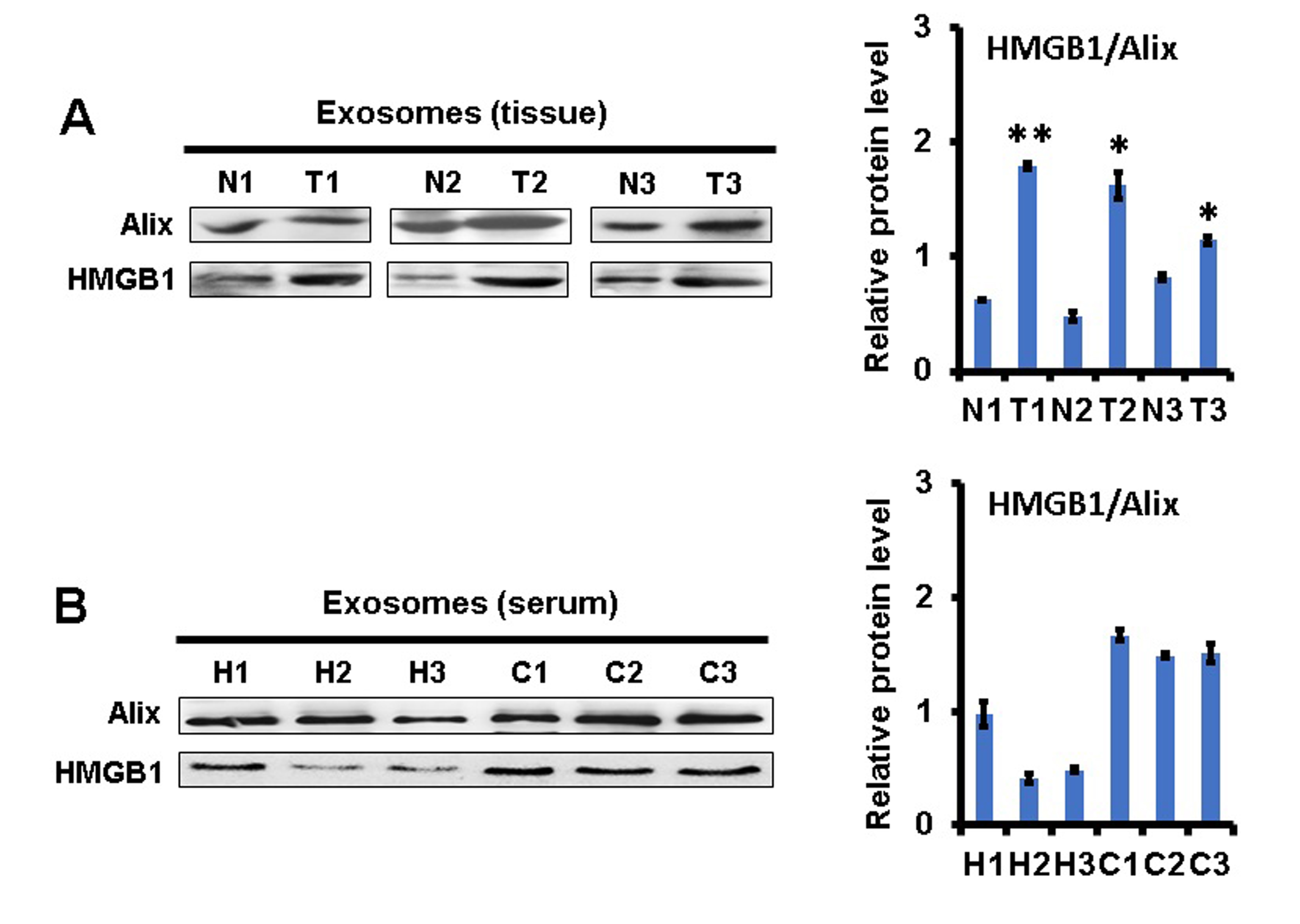

Supplement: Supplementary file 8 — Figure S7. Exosomal HMGB1 expression in tumor tissues and serum samples of gastric cancer patients. A. The expression of exosomal HMGB1 in the culture supernatants of tumor tissues and adjacent normal tissues (n=3) of gastric cancer patients was detected by using western blot. B. The expression of exosomal HMGB1 in the serum samples of healthy controls and gastric cancer patients (n=3) was detected by using western blot. (JPG 996 kb) [file 12943_2018_898_MOESM8_ESM.jpg]

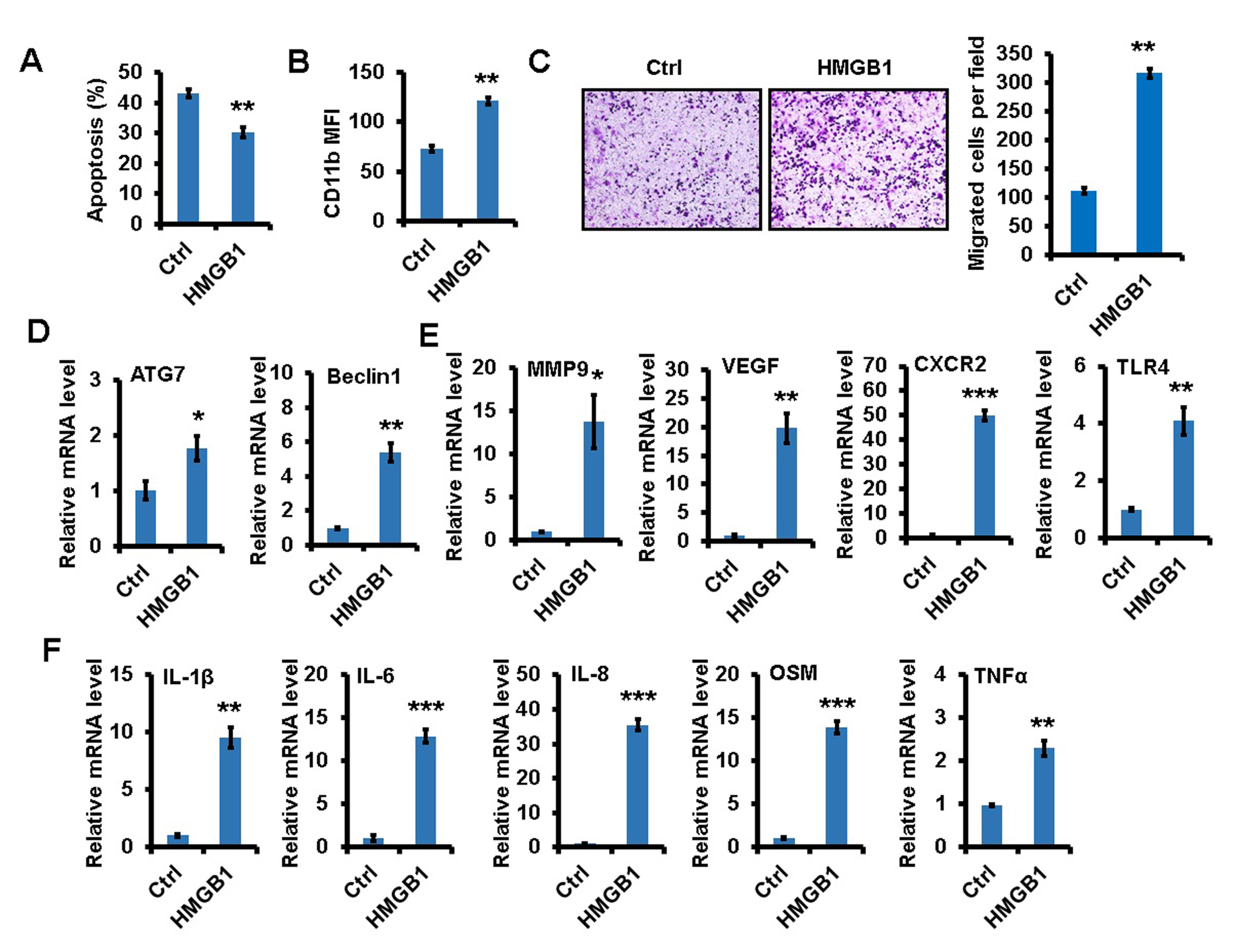

Supplement: Supplementary file 9 — Figure S8. Recombinant HMGB1 induced autophagy and promoted the activation of neutrophils. A. The percentage of apoptotic neutrophils following treatment with recombinant HMGB1 was determined by flow cytometric analyses. B. Flow cytometric analyses of CD11b expression in recombinant HMGB1-treated neutrophils. C. Transwell migration assays for gastric cancer cells after treatment with supernatant from recombinant HMGB1-treated neutrophils. D. The expression of ATG7 and BECN1 genes in recombinant HMGB1-treated neutrophils was measured by qRT-PCR. E. The expression of MMP-9, VEGF, CXCR2, and TLR4 genes in neutrophils treated with recombinant HMGB1 was determined by qRT-PCR. F. The expression of pro-inflammatory factors (IL-1β, IL-6, IL-8, OSM, and TNFα) in neutrophils treated with recombinant HMGB1 was measured by qRT-PCR. ***P<0.001, **P<0.01, and *P<0.05 compared to control. (JPG 1651 kb) [file 12943_2018_898_MOESM9_ESM.jpg]
