# Supplementary material for: Tumor-derived exosomes induce N2 polarization of neutrophils to promote gastric cancer cell migration
Source: Mol Cancer. 2018 Oct 6;17:146. doi: 10.1186/s12943-018-0898-6 (PMC6174070; doi:10.1186/s12943-018-0898-6)
Supplement: Supplementary file 7 — Table S3. The list of proteins identified in all the gastric cancer cells derived exosomes. (DOCX 18 kb) [file 12943_2018_898_MOESM7_ESM.docx]

**Table S3. The list of proteins identified in all the gastric cancer cells derived exosomes.**

| Gene name | Description |
| --- | --- |
| AGRN | Agrin |
| A2M | Alpha-2-macroglobulin |
| ACTN4 | Alpha-actinin-4 |
| APOA | Apolipoprotein A |
| APOE | Apolipoprotein E |
| APOH | Beta-2-glycoprotein 1 |
| [F5](http://www.baidu.com/link?url=DOmxcI9Du_C0TUYyR78tcR82tEtcpVCbAk6miErh_GVmEno-j4v4YyzaT0dBqXB2) | Coagulation factor V |
| COL18A1 | Collagen alpha-1 |
| COL18A2 | Collagen alpha-2 |
| C9 | Complement C9 |
| FASN | Fatty acid synthase |
| FN1 | Fibronectin |
| [LGALS3BP](http://www.baidu.com/link?url=GkPfrXVKKPik16WLT6eU3CtrlkGJV8gdLiOdlhKtlERtFr6kSbneySXF8Ju096LZ) | Galectin-3-binding protein |
| HSP90 | Heat shock protein 90kDa protein |
| HSP70 | Heat shock cognate 70 kDa protein |
| HMGB1 | High mobility group protein B1 |
| LAMA1 | Laminin subunit alpha 1 |
| NOTCH3 | Notch homolog 3 |
| PEDF | Pigment epithelium-derived factor |
| PLG | Plasminogen |
| PKM | Pyruvate kinase type M |
| RAB8A | Ras-related protein Rab-8A |
