# Supplementary material for: Tumor-derived exosomes induce N2 polarization of neutrophils to promote gastric cancer cell migration
Source: Mol Cancer. 2018 Oct 6;17:146. doi: 10.1186/s12943-018-0898-6 (PMC6174070; doi:10.1186/s12943-018-0898-6)
Supplement: Supplementary file 10 — Table S1. The sequences of primers for qPCR. (DOCX 18 kb) [file 12943_2018_898_MOESM10_ESM.docx]

**Table S1. The sequences of primers for qPCR**

| Gene | Sequence | Size (bp) | T_m_ (^o^C) |
| --- | --- | --- | --- |
| β-actin | F: 5'-CACGAAACTACCTTCAACTCC-3'  R: 5'-CATACTCCTGCTTGCTGATC-3' | 265 | 60 |
| IL-1β | F: 5'- TACGAATCTCCGACCACCA-3’  R: 5'- GGACCAGACATCACCAAGC-3’ | 256 | 60 |
| IL-6 | F: 5'-TACATCCTCGACGGCATCTC-3'  R: 5'-AGCTCTGGCTTGTTCCTCAC-3' | 252 | 61 |
| IL-8 | F: 5'-GCTCTGTGTGAAGGTGCAGTTT-3'  R: 5'-TTCTGTGTTGGCGCAGTGT-3' | 144 | 62 |
| TNFα | F: 5'-CCGAGTGACAAGCCTGTAGC-3'  R: 5'-AGGAGGTTGACCTTGGTCTG-3' | 493 | 57 |
| OSM | F: 5'-CACAGAGGACGCTGCTCAGT-3'  R: 5'-GCTGGTGTCCTGCATGAGA-3' | 143 | 60 |
| Mcl-1 | F: 5'-ACGGCCTTCCAAGGCAT-3'  R: 5'-TTGTTACGCCGTCGCTGA-3' | 103 | 63 |
| Fas | F: 5'-TCGGAGGATTGCTCAACAAC-3'  R: 5'-ATGATGCAGGCCTTCCAAGT-3' | 183 | 63 |
| ATG7 | F: 5’-GCCATGATGTCGTCTTCCTA-3'  R: 5’-CCGGTCTCTGGTTGAATCTC-3' | 299 | 60 |
| Beclin1 | F: 5’-TGATCCAGGAGCTGGAAGAC-3’  R: 5’-CACTGTGCCAGATGTGGAAG-3’ | 258 | 61 |
| MMP9 | F: 5’- ACGTCTTCCAGTACCGAGAG-3’  R: 5’- GGCACTGCAGGATGTCATAG-3’ | 126 | 60 |
| VEGF | F: 5’- CCATTGTGGAGGCAGAGAAA-3’  R: 5’- GATCAGGGAGAGAGAGATTGGA-3’ | 237 | 60 |
| CXCR2 | F: 5’-CAGCGACCCAGTCAGGATTTA-3’  R: 5’-ACCAGCATCACGAGGGAGTTT-3’ | 250 | 60 |
| TLR4 | F: 5’-GTCCTCAGTGTGCTTGTAGTATC-3’  R: 5’- CATTCCTTACCCAGTCCTCATC-3’ | 160 | 60 |
