# Supplementary material for: Tumor-derived exosomes induce N2 polarization of neutrophils to promote gastric cancer cell migration
Source: Mol Cancer. 2018 Oct 6;17:146. doi: 10.1186/s12943-018-0898-6 (PMC6174070; doi:10.1186/s12943-018-0898-6)
Supplement: Supplementary file 11 — Table S2. The target sequence of HMGB1 siRNA. (DOCX 15 kb) [file 12943_2018_898_MOESM11_ESM.docx]

**Table S2. The target sequence of HMGB1 siRNA**

| siRNA | Target sequence |
| --- | --- |
| si-scramble | UUCUCCGAACGUGUCACGUAA |
| si-HMGB1 | CCGUUAUGAAAGAGAAAUGAAU |
